# Mesoscopic Model of Actin-Based Propulsion

**DOI:** 10.1371/journal.pcbi.1002764

**Published:** 2012-11-01

**Authors:** Jie Zhu, Alex Mogilner

**Affiliations:** Department of Neurobiology, Physiology and Behavior and Department of Mathematics, University of California, Davis, Davis, California United States of America; Northeastern University, United States of America

## Abstract

Two theoretical models dominate current understanding of actin-based propulsion: microscopic polymerization ratchet model predicts that growing and writhing actin filaments generate forces and movements, while macroscopic elastic propulsion model suggests that deformation and stress of growing actin gel are responsible for the propulsion. We examine both experimentally and computationally the 2D movement of ellipsoidal beads propelled by actin tails and show that neither of the two models can explain the observed bistability of the orientation of the beads. To explain the data, we develop a 2D hybrid mesoscopic model by reconciling these two models such that individual actin filaments undergoing nucleation, elongation, attachment, detachment and capping are embedded into the boundary of a node-spring viscoelastic network representing the macroscopic actin gel. Stochastic simulations of this ‘in silico’ actin network show that the combined effects of the macroscopic elastic deformation and microscopic ratchets can explain the observed bistable orientation of the actin-propelled ellipsoidal beads. To test the theory further, we analyze observed distribution of the curvatures of the trajectories and show that the hybrid model's predictions fit the data. Finally, we demonstrate that the model can explain both concave-up and concave-down force-velocity relations for growing actin networks depending on the characteristic time scale and network recoil. To summarize, we propose that both microscopic polymerization ratchets and macroscopic stresses of the deformable actin network are responsible for the force and movement generation.

## Author Summary

There are two major ideas about how actin networks generate force against an obstacle: one is that the force comes directly from the elongation and bending of individual actin filaments against the surface of the obstacle; the other is that a growing actin gel can build up stress around the obstacle to squeeze it forward. Neither of the two models can explain why actin-propelled ellipsoidal beads move with equal bias toward long- and short-axes. We propose a hybrid model by combining those two ideas so that individual actin filaments are embedded into the boundary of a deformable actin gel. Simulations of this model show that the combined effects of pushing from individual filaments and squeezing from the actin network explain the observed bi-orientation of ellipsoidal beads as well as the curvature of trajectories of spherical beads and the force-velocity relation of actin networks.

## Introduction

Cell migration is a fundamental phenomenon underlying wound healing and morphogenesis [1]. The first step of migration is protrusion – actin-based extension of the cell's leading edge [2]. Lamellipodial motility [3] and intracellular motility of the bacterium *Listeria monocytogenes* [4] are two prominent model systems that in the past decades have added considerably to our understanding of the protrusion based on growth of actin networks. These *in vivo* systems are complemented by *in vitro* assays using plastic beads [5] and lipid vesicles [6] that, when coated with actin accessory proteins, move much the same way as the *Listeria* pathogen.

**Figure 1:**
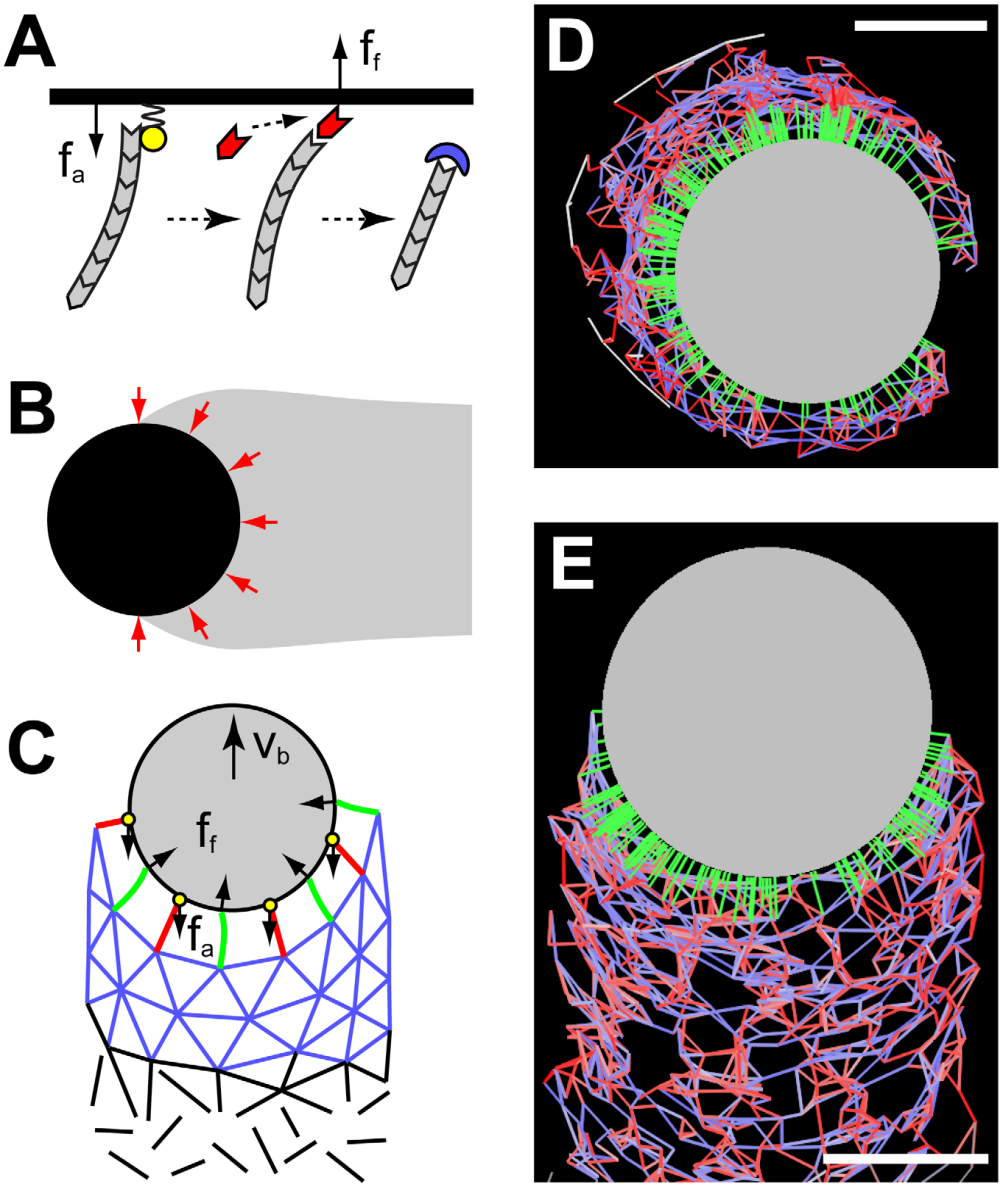
Schematics of the models. (A) Tethered ratchet model. Actin filaments (gray) can attach to the obstacle surface (black line) via attachment sites (yellow) and exert pulling forces (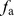). Detached filaments can elongate by assembling actin monomers (red) onto their barbed ends and exert pushing forces (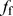) via a Brownian ratchet mechanism. Detached filaments are eventually capped by capping proteins (blue) and stop interacting with the obstacle. (B) Elastic theory. An elastic actin network (gray) propels a curved obstacle (black) with ‘squeezing’ forces (red arrows). (C) Hybrid model incorporating both discrete filaments (green lines: free filaments; red lines: attached filaments; yellow circles: attachment sites) and deformable network (blue lines), which is treated as a node-spring meshwork. Filaments are created along the surface of the bead (gray) and immediately anchor to the network in an undeformed state. Filaments exert forces on the bead as well as the network. The network is then deformed in response to the forces from the filaments. The springs of the network can be ruptured by a high stretching force. The network's nodes, together with connected springs, are removed from the network at a constant rate to represent the disassembly of the network (black lines). (D–E) Simulation snapshots of an actin-propelled bead (gray circle) during (D) symmetry breaking and (E) steady movement. Green lines: interacting filaments. Blue lines: stretched network springs. Red lines: compressed network springs. Bars: 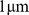.

Here we examine computationally the mechanics of growing actin networks. This problem has a long history starting from applying thermodynamics to understand the origin of a single filament's polymerization force [7]. The notion of polymerization ratchet led to the derivation of an exponential force-velocity relation (Figure S1 in Text S1) for a rigid filament growing against a diffusing obstacle [8]. Then, elastic polymerization ratchet model [9] was proposed for flexible actin filaments. This model evolved into tethered ratchet theory, in which a dynamic balance between surface-pushing growing filaments and motion-resisting attached filaments (Figure 1A) governs the protrusion [10]. These early theories considered independent single filaments. However, actin filaments do not grow individually, but evolve interdependently as a network by branching sideways from each other [11]. Mathematical treatments and computer simulations of branching and nucleation [12], [13] of filaments growing against an opposing force, which treated the dendritic actin network as a mechanically rigid body, predicted various force-velocity relations. Those ranged from concave-down (velocity of protrusion being insensitive to the load up to a threshold and plunging to a stall at a critical opposing force) to concave-up (more or less exponential decrease of the velocity with the growing load) relations (see Figure S1 in Text S1). These theoretical efforts culminated in detailed agent-based three-dimensional (3D) models of growing networks of rigid filaments propelling *Listeria* pathogen [14], [15].

In parallel to these microscopic theories, macroscopic elastic propulsion model [16], [17] suggested that the curved surface of the pathogen is not merely pushed, but squeezed forward by an elastic stress. This stress is developed from the stretching of the outer layer of actin gel by the growth of the gel near the inner surface (Figure 1B). This model treated the actin network as an isotropic elastic continuum and did not explicitly consider the microscopic mechanism of force generation at the surface. As a result, a concave-up force-velocity relation for the actin-propelled spherical bead was derived [18], predicting an initial rapid decay with opposing force followed by a region of slower decay of velocity. This prediction was confirmed by using a cantilever setup for beads coated with the actin polymerization activator N-WASP and moving in a pure-protein medium [18]. On the other hand, when the force-velocity relation of an actin network growing against a flat surface was measured using the cantilever method, it was found that the growth velocity was constant at small forces but dropped rapidly at higher forces [19] as predicted by some microscopic ratchet theories.

Note that the widely used terminology could be confusing as the elastic propulsion theory is sometimes called mesoscopic rather than macroscopic. Both terms are justified: the macroscopic mechanics is described using continuum theory, but an actin layer of a few microns thin is certainly a mesoscopic system. The model we present is mesoscopic in the sense that it spans from the microscopic level of individual filaments to the macroscopic level of continuous description of an actin gel. The model is also hybrid because it takes into account both local discrete forces and global network stress. We will mostly use the term “hybrid” throughout the paper.

The first simple attempt to use hybrid modeling of the lamellipodial edge was recently made in [20], where the actin network was divided into a semiflexible region near the membrane and a gel-like region at the back. Near the membrane, semiflexible filaments are assumed to produce entropic forces against both the membrane and the gel. In the back, the viscous gel deforms in response to stresses both from frontal filaments and internal contractions, causing retrograde flow. Because the semiflexible region is assumed to be supported by the gel region, the moving speed of the membrane is determined by the coupling between the two regions. This model was able to reproduce both concave-up and concave-down shapes of the force-velocity relation. Since this model considered only a one-dimensional strip of actin gel, it did not address the effects of surface geometry.

Besides the force-velocity relation, the non-zero curvatures of the trajectories of motile objects [21] is another important observable. A pioneering microscopic ratchet-based model, which investigates how randomly distributed actin filaments propel a cigar-shaped pathogen, predicted that the resultant bacterial trajectories have curvature values following a Gaussian distribution with zero mean [22]. This conclusion was challenged by a number of studies. One of them showed helical movements that were explained as a result of a non-vanishing torque that arises from a persistent actin-induced off-center force [23]. Another study did not result in helical paths of beads, but rather showed a highly varying curvature of trajectories which has a Gaussian distribution, albeit with a sharp peak at zero curvature [24]. In contrast, a third study indicated that the distribution of the curvatures of trajectories deviated significantly from Gaussian, which was explained by a cooperative breaking of filaments tethered to the bead [25]. All theories used to explain these experiments were microscopic; elastic propulsion model was never applied to these phenomena.

Below, we describe observations of ellipsoidal, rather than spherical, beads that cannot be explained by either microscopic or macroscopic model. This, as well as the complex force-velocity relation and curvature distribution described above, hints that perhaps a hybrid model with individual actin filaments pushing from the surface of a macroscopic deformable actin gel can explain the experiments better. Recent experiments and theory [26], [27] demonstrated that disassembly and breaking of the actin gel are as important as the elastic deformations in generating propulsion. Therefore, we developed a model of a node-spring viscoelastic network representing the actin gel with individual pushing and pulling filaments embedded into the network boundary. Simulations of this *in silico* hybrid network showed that the combined effects of the macroscopic viscoelastic deformation and microscopic ratchets can explain both concave-up and concave-down force-velocity relations for growing actin networks, bistable orientation of the actin-propelled ellipsoidal beads, and peculiar curvature distributions for the actin-propelled trajectories of the beads.

## Results

### Computational Model

We developed a two-dimensional (2D) simplification of a 3D hybrid model (Figure 1C), which incorporates both arrays of dynamic actin filaments at the surface-tail interface and the bulk deformable actin gel behind the interface. Filament arrays are embedded into the boundary of the deformable actin gel, which is coarse-grained into a network of nodes interconnected by elastic springs. Individual filament arrays at the surface-tail interface switch between pushing the obstacle surface and attaching to it. The existing filaments are constantly becoming a part of the network and dynamically expanding the actin gel, while nascent filament arrays are created around the surface via a mixture of nucleation and branching processes. The actin network undergoes disassembly, which is treated by removing the nodes and springs at a constant rate, as well as by rupturing crosslinks at a critical stretching force. The deformations of the network as well as the elastic filament forces cause both translational and rotational motion of the bead. The model reproduces the steady motion of beads propelled by treadmilling actin tails behind the beads (Video S1). Further details about the model assumptions, equations, numerical simulations and model parameters are described in the Materials and Methods and Text S1.

### Orientation of Ellipsoidal Beads

**Figure 2:**
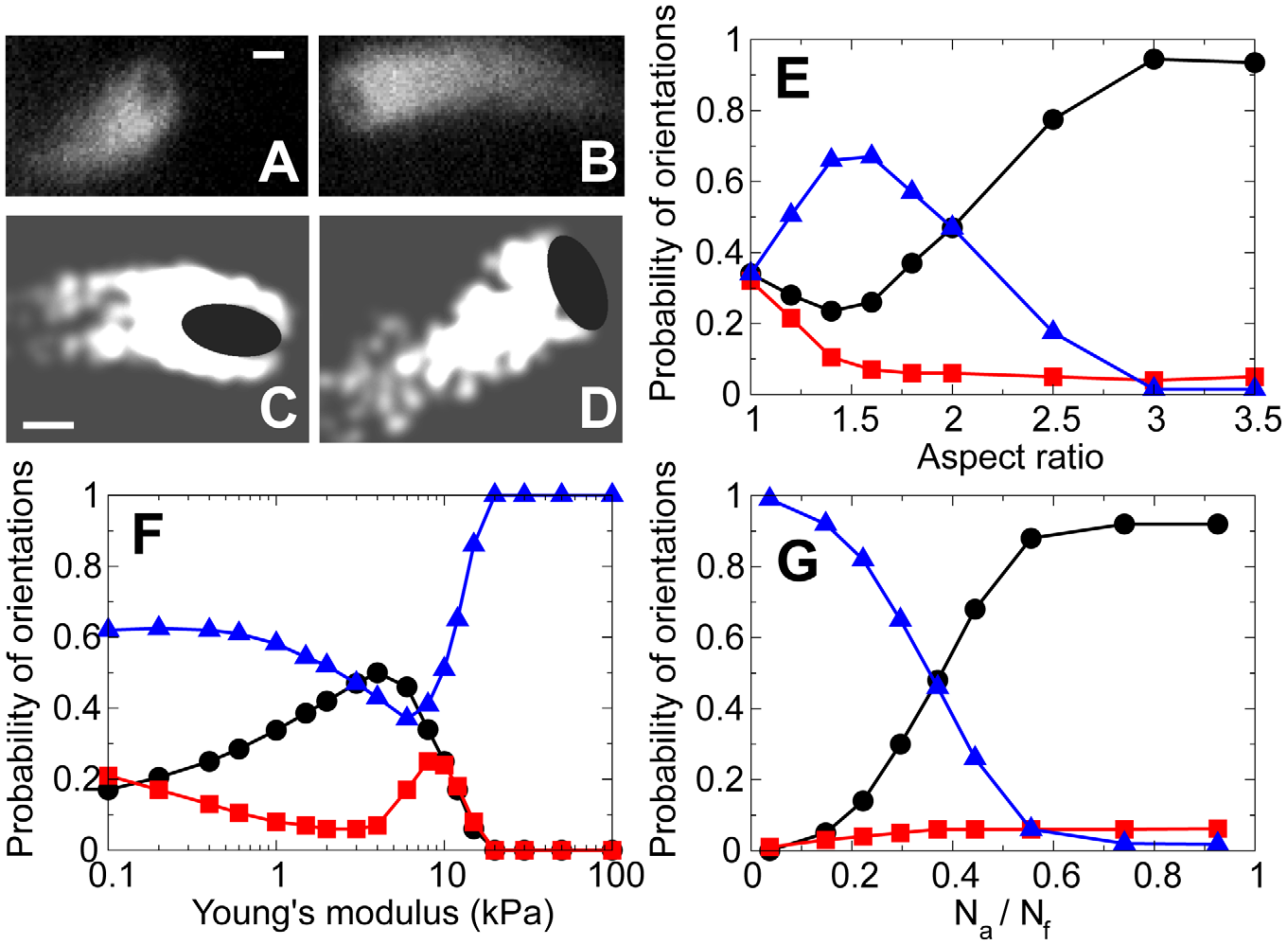
Motion of actin-propelled ellipsoidal beads. (A–B) Fluorescent images show actin tails of the motile beads. The dark ellipsoidal shapes at the fronts of the tails illustrate bead's propulsion along its (A) long-axis and (B) short-axis. The detailed statistics of phase contrast images reported in [28] confirm that roughly halves of the beads move in each orientation. Bars: 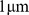. (C–D) Simulation snapshots of the same bead moving along its (C) long-axis and (D) short-axis at different time moments. Black circle: bead. White: actin networks with each node being a Gaussian-blurred dot of 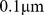 in decay width. Bars: 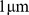. (E–G) Probability distribution of bead's orientation as a function of (E) bead's aspect ratio, (F) Young's modulus of actin networks, and (G) ratio of the numbers of attached and pushing filaments. Black circles: bead moves along the long-axis (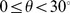). Red squares: bead moves at a skewed orientation (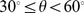). Blue triangles: bead moves along the short-axis (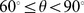).

Recently, with our experimental collaborators, we reported observations of the ellipsoidal beads that were uniformly coated with an actin assembly-inducing protein (ActA) [28] and moved in the plane between two parallel coverslips (see the Materials and Methods below). Surprisingly, roughly half of the time the beads moved along their long axes, and another half – along their short axes (Figure 2, A and B), with infrequent switches between these orientations.

To see whether the two existing models of actin propulsion can explain this result, we simulated the motion of actin-propelled ellipsoidal beads as described in the Materials and Methods. Elastic theory predicts that squeezing of an ellipsoidal bead introduces a torque orienting the bead with its long axis parallel to the actin tail (see Figure S2 and Figure S6 in Text S1). In agreement with this prediction, when we decreased the autocatalytic branching of actin and attachment forces, so that the actin gel exerted almost uniform normal stress on the bead surface, the model resulted in a propulsion along the bead's long axis (Video S2). On the other hand, when we simulated a network of rigid branching filaments pushing the bead, the propulsion was always along the short axis, so the bead moved sideways (Video S3). This change in the preferred orientation is caused by a subtle bias in how the actin network spreads along the bead surface: if the bead's orientation is skewed relative to the actin tail's axis, filament branching are more likely to happen near the tail-facing flatter surface where there is a higher number of existing filaments. As a result, more filaments push the bead sideways from the actin tail, shifting the filament-contacting region from the curved surface to the flatter one. Eventually, most filaments branch against the flatter part of the surface, orienting the bead with its long axis normal to the tail axis (see Figure S7 and detailed calculations in Text S1).

Thus, the elastic propulsion model predicts that beads only move along their long axes, while microscopic ratchet model predicts that beads only move along their short axes, and neither model can explain the observation. In contrast, the full hybrid model predicts that the bead can move in both orientations due to the combination of the elastic squeezing and the geometric spreading of actin and switch infrequently between them (Video S1, Figure 2, C and D, Figures S5 and Figure S8 in Text S1), in agreement with the observation (Figure 2, A and B). For more insight into this phenomenon and to generate predictions for experiment, we investigated numerically how the fraction of beads moving with a certain orientation depends on the geometric, mechanical and kinetic parameters.

#### Bead's aspect ratio

The simulation results of the effects of a bead's aspect ratio (at constant area of the bead) on its orientation are shown in Figure 2E. Beads with aspect ratios greater than 2 are more likely to move along their long axes, whereas movement along the short axes arises in beads with aspect ratios smaller than 2. For a spherical bead, motion has no preference along any axis (in this case the initial direction of axes is arbitrarily defined), as expected. This can be qualitatively explained as follows: for a highly elongated bead, the elastic squeezing action from the sides is greater, plus the actin network is more likely to rupture near the highly curved poles of the bead, which together orients the bead and tail axes in parallel. For a less elongated bead, the elastic torque becomes smaller, while the geometric effect spreading actin along flatter side of the bead persists. In Text S1, we show that the above results can be explained by the nonlinear dependence of the overall rotation on the aspect ratio of beads.

#### Network's stiffness

We vary the Young's modulus of the actin network by varying the spring constant in our model as described in Text S1. The effects of network stiffness on the orientation of the bead with aspect ratio of 2 is shown in Figure 2F. We find that when the actin gel is very soft (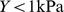) or very stiff (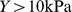), the bead prefers moving sideways, along its short-axis. On the other hand, when the network has an intermediate stiffness (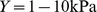), the bead can move along either axis with similar probability. Indeed, for a very stiff network, the elastic deformation becomes negligible. Pushing and reorientation of individual filaments determine the bead's motion along its short-axis, consistent with the microscopic model. For a network with intermediate stiffness, the network squeezing effect, which align the bead to move along its long-axis, is comparable to the pushing and reorientation effects of the filaments, so the bead has similar chances to choose either orientations. For a very soft network, the network is highly deformable and thus is less likely to provide enough squeezing force to align the bead to move along its long-axis. In Text S1, we also show the results for beads with different aspect ratios. As beads' aspect ratio increases from 1.5 to 2.5, the preferred orientation shifts from short to long axis if the network has an intermediate stiffness (see Figure S9 in Text S1). For much softer or stiffer networks, beads always prefer moving along their short axes.

#### Effect of filament attachments

The effect of the ratio of the number of attached to the number of pushing filaments, 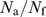, on bead's orientation is shown in Figure 2G. As 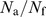 ratio increases, the bead is more likely to move along its long-axis: when more attached filaments pull on the bead, the bead moves slower and have a denser network around it, and the elastic squeezing effect is strong. At a low 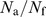 ratio, most filaments are pushing and few are pulling. The bead moves fast and tends to leave the network behind, so the squeezing from the sides becomes small, elastic effect is negligible, so the bead moves along its short axis. At 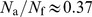, the bead has similar chances to move with either orientation. Although the attachment dependence of orientations depends on the balance between torques from free and attached filaments, it is challenging to find a simple analytical formula. It is because the nonlinear actin-remodeling-induced turning of the actin tail also plays an important role (see Text S1), which impedes a clearer physical picture of how 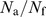 affects the orientation of beads.

### Trajectory of Actin-Propelled Spherical Beads

**Figure 3:**
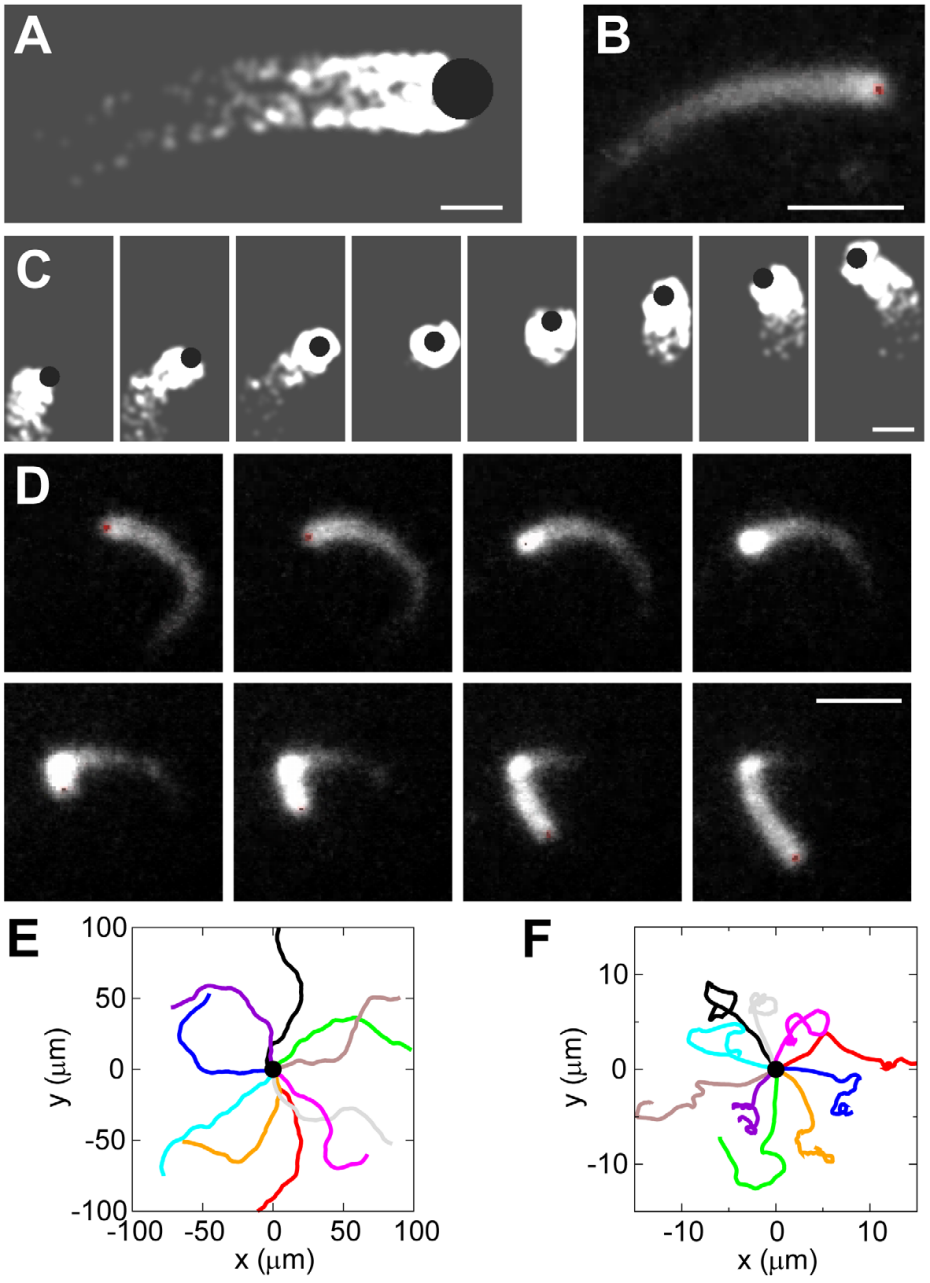
Trajectories of actin-propelled spherical beads. (A) Simulation snapshot of the hybrid model. Black circle: bead. White: actin network. Bar: 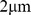. (B) Fluorescent images of actin tails behind 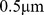 beads. Bar: 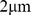. (C) Simulation snapshots of bead with 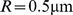 and 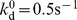. Time interval is 100 s. Bar: 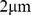. (D) Sequential snapshots of an observed bead that is temporarily trapped by its actin tail. Time interval is 20 s. Bar: 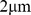. Courtesy of J. Theriot's lab. (E–F) Ten simulated bead's trajectories (colored lines) starting from the same origin (black dot) with (B) default values of parameters (see Table S1 in Text S1) and (C) same as (B) but with low value of detachment rate 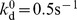. Each simulation represents 3600 s in real time.

To further test the hybrid model, we simulated the motion of actin-propelled spherical beads (Figure 3, A and C). We recorded the 2D ‘*in silico*’ trajectories of the beads and compared them to the experimental observations (see the Materials and Methods). We examined two possible mechanisms for the nucleation of new filaments: autocatalytic branching and spontaneous nucleation. We found that each mechanism alone does not produce the observed motion of the bead (see Video S4 and Video S5). Only a combination of the two mechanisms leads to realistic motion of the bead (see Video S6 and details in Text S1). Note that the trajectories are easy to visualize by looking at the actin tails that represent the most recent parts of the trajectories, see Figure 3, B and D). Our typical simulation results (Figure 3, A and E, Video S7) illustrate that in general the trajectories are mildly curved, as observed in some cases experimentally (Figure 3B). However, in other cases the experimental observations (Figure 3D) show that once in a while the beads stop, get surrounded by a dense actin ‘cloud’, and then break through the cloud and resume movement in a new direction.

Indeed, the model predicts that when the detachment rate of actin filaments becomes low and a greater fraction of filaments is attached to the bead surface, beads start to have pulsatory motion due to temporary entrapment by the actin gel (Figure 3C and Video S8), which occurs frequently in this regime. The explanation is that when filaments detach rapidly and thus do not generate great pulling forces, beads move quickly and can hardly be trapped, but at low detachment rate, beads slow down significantly by the strong pulling forces, which increases their chances to be trapped into the actin gel. Both our simulations and observations from our collaborators show that beads often make sharp turns during their escapement from the surrounding actin gel (Figure 3, C and D), causing the switching between the low- and high-curvature trajectories. As a result, the trajectories show spatially separated segments of low and high curvatures (Figure 3F).

**Figure 4:**
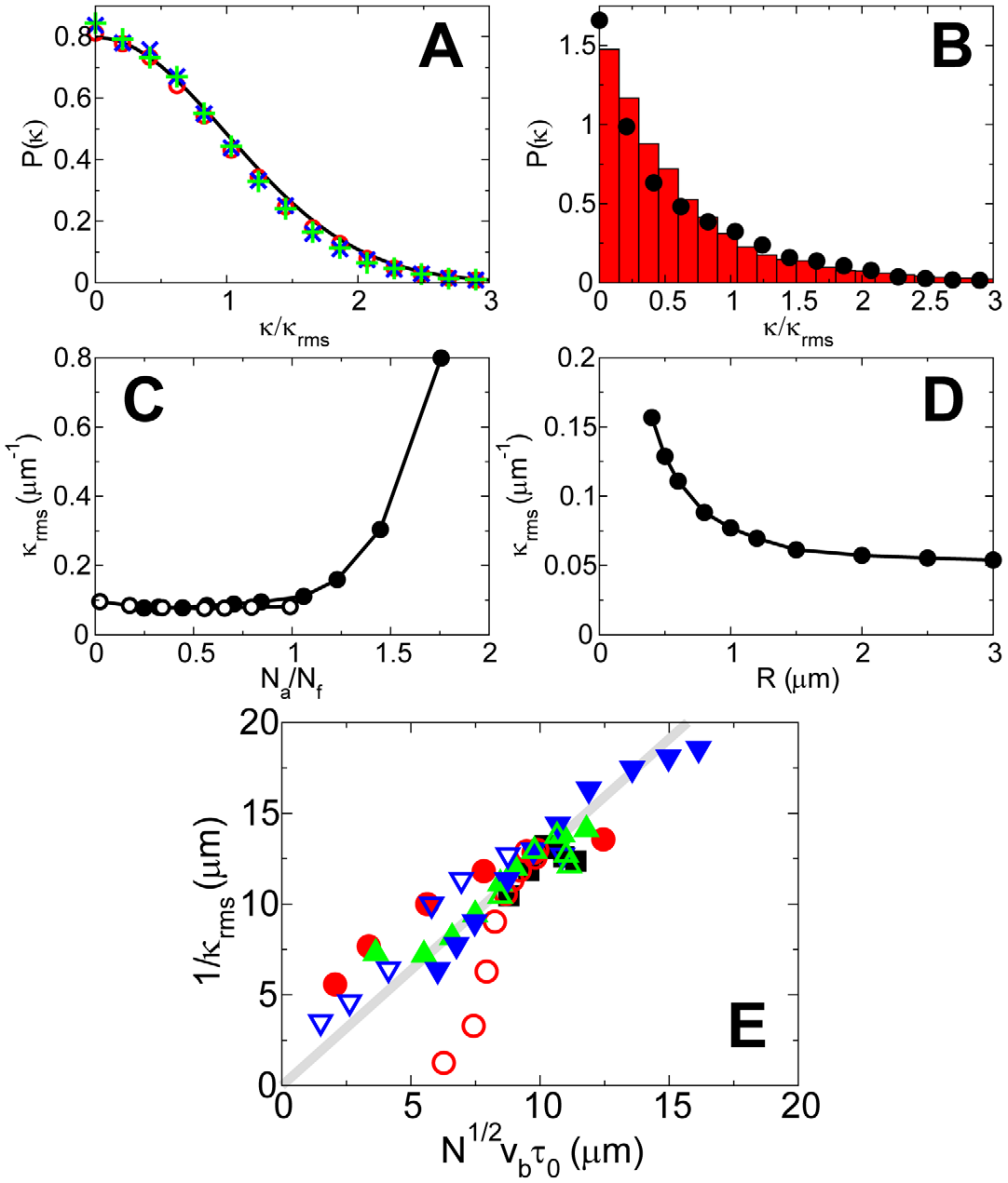
Trajectory curvature of actin-propelled spherical beads. (A) Probability distribution of the normalized trajectory curvature for default values of parameters (open red circles), twice the value of attached to pushing filament ratio (green pluses) and twice the bead radius (dotted line), compared to a Gaussian distribution (solid black line). (B) Probability distribution of the normalized trajectory curvature with 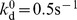 (circles) compared with experimental results (bars). (C) Dependence of the root-mean-square curvature on the attached to pushing filament ratio varied by varying 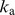 (open circles) and 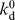 (solid circles). (D) Dependence of the root-mean-square curvature on the bead's radius. (E) Dependence of the inverse root-mean-square curvature on 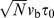. Solid gray line: analytical prediction. Symbols: values of 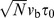 changed by varying 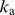 (solid black square), 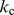 (solid red circle), 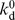 (open red circle), 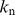 (solid green up-triangle), 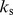 (open green up-triangle), 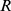 (solid blue down-triangle) and 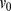 (open blue down-triangle).

To obtain the distribution of the curvatures of the trajectories, we smoothed the simulated bead's trajectory to remove the high frequency noises and calculated (see Text S1 for details) that the curvature distribution is close to Gaussian (Figure 4A) for fast-moving beads in the wide range of parameters. This indicates that the turning of the fast-moving bead is likely to be driven by random events in the protruding actin network.

When the detachment rate is low, we find that the curvature distribution becomes sharply peaked at zero (Figure 4B), in agreement with both our observation (Figure 4B) and previous results [24]. Since the low- and high-curvature trajectories are typically separated in this regime, this sharp peak near zero is due to bead moving in a rapid-and-smooth fashion, while the slowly decreasing distribution at higher curvatures is caused by bead moving in a slow-and-jagged fashion. Furthermore, we find that the distribution is close to a Gaussian at higher curvature, indicating that the highly curved segments of trajectories are also likely to be caused by the random fluctuations in the actin network.

We found that the predicted characteristic value of the root-mean-square curvature, 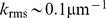 (Figure 4C), is of the same order of magnitude as our observations (Figure S17 in Text S1) and available measurements [4], [24], [25]. We investigated how the filament attachments affect the value of 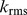 (Figure 4C) and found that 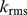 is insensitive to 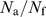 for 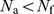. However, the curvature increases rapidly with 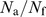 for 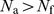, consistent with the idea that excessive attached filaments cause frequent trapping of the bead leading to highly curved trajectories.

We also studied how the bead radius, 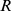, affects 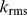 (Figure 4D) and found that decreases as the bead size increases. This result is in agreement with the experimental observations reported in [4], [25]. Interestingly, this results is also consistent with our experimental observation on the orientation-dependent turning of the trajectories of ellipsoidal beads (Figure S17 in Text S1): ellipsoidal beads moving along their long-axes are less likely to keep their current direction of motion comparing to those moving along their short-axes. A possible interpretation is that the former are mostly pushed at their sharp ends where the radius of curvature is low. Similar to a spherical bead with small 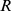, this will lead to a high 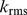 in the trajectory and thus will be less likely for the bead to keep the current direction of motion. Together, the above results can be explained as follows: larger beads are propelled by a greater number of filaments, so relative fluctuations in the actin network go down and thus the beads fluctuate less in their motion. These findings suggest that the fluctuation in the number of actin filaments is likely the factor determining the curvature, so we developed a simple model to understand and test such mechanism.

Two possible mechanisms may contribute to the turning of beads' trajectory: turning induced by elastic and ratchet torque, and turning induced by actin tail-reorientation (see Text S1). Because of the symmetry of the spherical bead, the torque-induced rotation found in the ellipsoidal beads is negligible. Our simulations also confirm that a micron-sized spherical bead rarely rotates about its center during its motion. Therefore, the re-orientation of the tail along the bead surface is likely to be the main cause of the trajectory turning. Thus, we consider a simplistic model in which a bead of radius 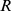 is propelled by 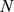 randomly distributed filaments at its rear, so the filament number difference between the left and right sides of the bead is on the order of 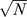. In other words, 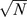 out of 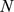 filaments tend to push the bead off the current direction by an angle 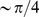 while the rest tend to push along the current direction of motion. The change in the direction of motion is expected to be 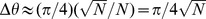. The typical time 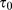 over which the directional bias persists is the turn-over time of the actin network, which we estimate in Text S1. Then, the typical angular velocity of the turning is 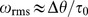, and the root-mean-square value of the curvature is 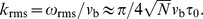 One thus expects a linear relation between 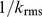 and 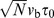 with a slope of 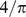. To test whether this simple conclusion is correct, we used simulations of the hybrid model to obtain the values of 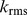, 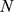, 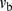 and 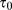. We plotted the simulation results for 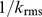 as a function of 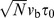 for various values of attachment, detachment, capping and nucleation rates, as well as of actin gel elastic constant, together with the predicted linear relation, and found very good agreement except for low values of the detachment rate (see Figure 4E, Figure S10 and Figure S11 in Text S1). The higher-than-expected values of 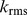 obtained from the simulations with low detachment rates are caused by the entrapment of beads into the actin gel, as mentioned above. Thus, macroscopic elastic effects influence the trajectory only in the limiting case of too many attached filaments. Otherwise, stochastic microscopic filament-ratchets are responsible for the curvature of trajectories.

Note that in contrast to our results, a non-Gaussian distribution of the curvatures of trajectories of the beads was observed in [25]. According to the model in [25], the torque balance alone determines the turning of the bead, while in our model both torque and redistribution of actin around the bead determine the trajectory. This difference suggests that the redistribution of actin probably does not play an important role in the experiments in [25]. One possibility is that the actin tail always interacts with a fixed side of the bead in these experiments, which can result from an asymmetric coating of the bead surface by the actin-nucleation promoting factors. Also note that the autocorrelation function of the simulated curvature of trajectories always decays rapidly at a sub-micron distance (see Figure S12 and details in Text S1). This result differs from the observed long-range correlation of about 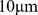 [24], which is possibly caused by additional long-ranged bias in the actin network near the bead-tail interface.

### Force-Velocity Relation of Actin Networks

**Figure 5:**
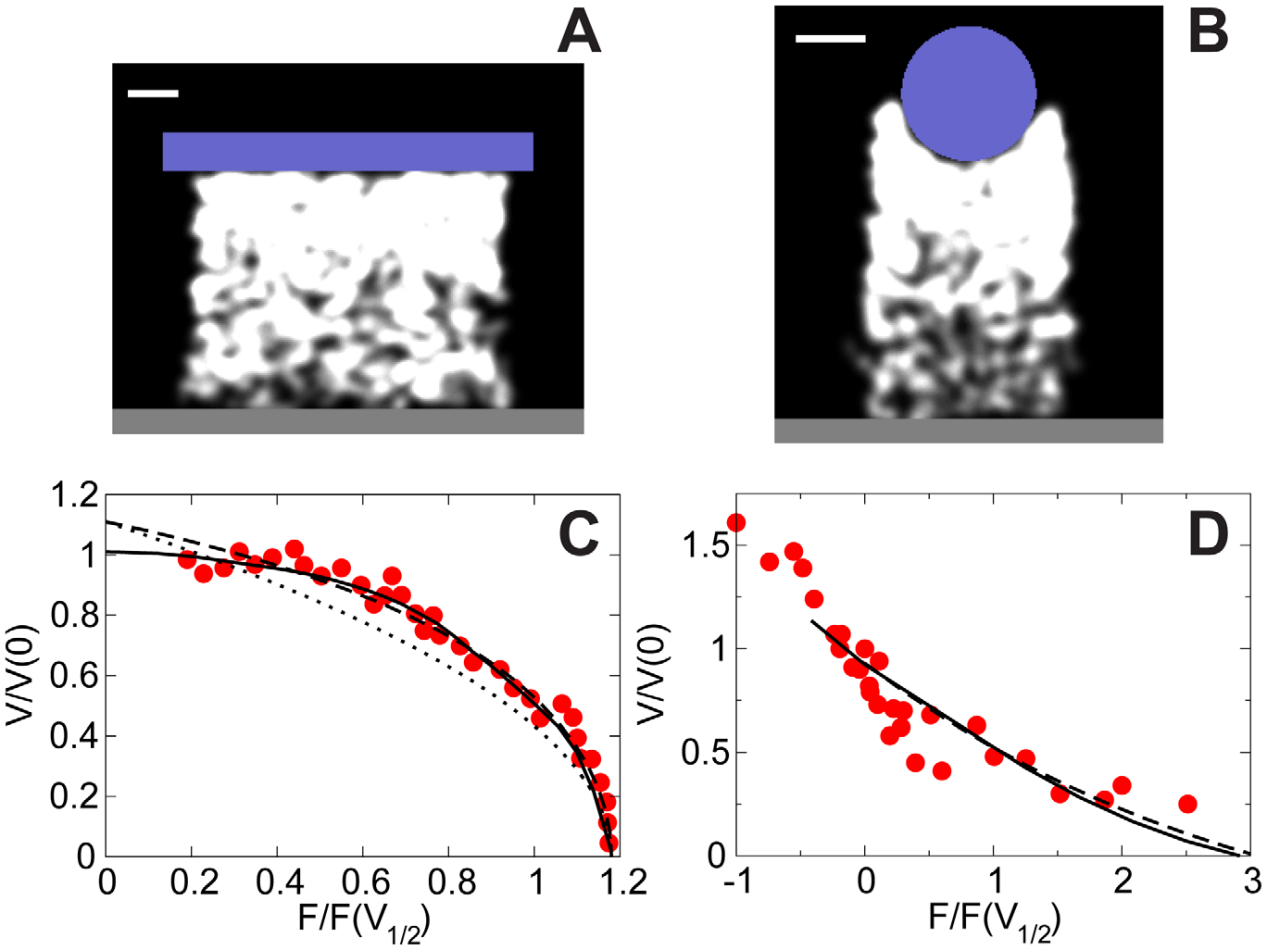
Simulated force-velocity relation of actin networks. (A–B) Snapshots of hybrid model simulations. Blue: obstacles. White: actin networks. Dark gray: rigid substrate. Bars: 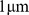. (A) Actin network grows continuously against a flat cantilever with force being proportional to the deflection. (B) Actin network grows against a spherical bead, with force being clamped for each velocity measurement. (C–D) Simulated force-velocity relation compared with the data. (C) Computational results corresponding to the setup in (A). Red circles: experimental data from [19]. Solid line: hybrid model simulation. Dashed line: prediction of the 1D theory in Text S1. Dotted line: approximate analytical formula 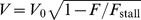. (D) Computational results corresponding to the setup in (B). Red circles: experimental data from [18]. Solid line: hybrid model. Dashed line: prediction of the 1D theory in Text S1.

We simulated growth of an actin pedestal against flat elastic cantilever and force-clamped spherical bead, as in experiments [18], [19], respectively (Video S9 and Video S10). The hybrid model in these cases was used as described above, with the following differences: 1) We first generated undeformed node-spring pedestal underneath the surface to be pushed. 2) All actin network nodes were free to be positioned by the force balances (the nodes in the network did not become immobile when they were more than a few microns away from the surface) except at the very bottom. The layer of the nodes at the very bottom was immobilized. 3) The motion of the cantilever or bead was determined by the balance between the pushing/pulling forces from the filaments touching the surface and either a) the elastic restoring force from the cantilever proportional to cantilever's deflection, or b) clumped force from the bead. The speed of the cantilever or bead, 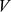, was then obtained by dividing the displacement increment of the surface by the time interval. Calibration of the model in these numerical experiments is described in Text S1. Simulation snapshots are shown in Figure 5, A and B and Figure S16 in Text S1.

The simulated force-velocity relation predicted by the hybrid model for the flat cantilever is compared to the experimental data [19] in Figure 5C. We scale the cantilever force 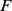 by 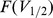, which is the force at half of the maximum cantilever speed and scale 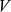 to best match the rest of the data. The prediction agrees very well with the observed concave-down force-velocity relation. To quantitatively understand this result, we develop an analytical 1D theory in Text S1 and find that continuing reduction of the network stiffness due to the network disassembly during a long time of the experiment plays an important role in the shape of the force-velocity relation. A network undergoing significant disassembly in the aged gel sections recoils under a high load, reducing both net protrusion rate of the actin network pushing the cantilever and the maximum force that the network can sustain. These factors cause the rapid downturn in the force-velocity relation. Our 1D analytical result (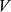 can be approximated as 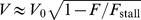 in relevant parameter range) is shown in Figure 5C and is in very good agreement with both experimental data and simulation of the 2D hybrid model.

We then used the hybrid model to simulate the force-velocity relation for the force-clamped bead. In this case, the force-velocity relation is concave-up, in good agreement with the observations [18] (Figure 5D, Figure S15 in Text S1). Qualitative explanation for this shape is that the velocities in this experiment were measured on a minute time scale before the network significantly disassembles (over a few minutes). Therefore, the network's recoil is negligible in this case and the force-velocity relation is similar to that of individual filaments. From our 1D calculation for 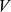 under a constant load 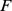 (see Text S1), we find 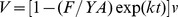, where 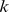 is proportional to the disassembly rate constant of the network and 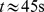 is the age of the network when 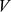 is measured in our simulations, and 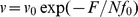 is the average velocity of 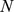 individual filaments. This analytical result is also shown in Figure 5D, in very good agreement with the simulation results of the hybrid model.

To investigate the effect of the filament attachments to the surface on the force-velocity relations, we varied the value of the attachment rate to change the ratio of the number of attached to the number of pushing filaments, 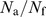. The simulated force-velocity relations for different ratios are shown in Figure S13 in Text S1. For both cantilever and force-clamped experiment, we find that increasing the fraction of attached filaments decreases both velocity and stall force without changing the qualitative shape of the force-velocity curve, consistent with the idea that attached filaments counteract the pushing filaments. Finally, to confirm that it is the actin dynamics rather than the shape of the surface that determines the force-velocity relation, we swapped the shapes of the flat cantilever and round bead used in the two experiments. We considered two cases: a slow-growing actin network against a curved surface of a cantilever, and a fast-growing actin network against a flat force-clamped object. The simulation results shown in Figure S13 and Figure S15 in Text S1 illustrate that the force-velocity relations in both experiments remain qualitatively the same (concave-down and concave-up, respectively). Therefore, the shape of the surface does not appear to affect the overall shape of the force-velocity relation.

## Discussion

Complexity of the relation between geometry of the curved surface, molecular pathways of actin polymerization against this surface and resulting force [29] indicates that the actin-based force-generation is a multi-scale phenomenon, understanding of which requires a combination of macroscopic and microscopic mechanisms. We developed such hybrid model of the actin network growing and pushing against rigid surfaces, in which actin filaments interacting directly with the surface are treated as tethered-ratchet filaments, while other filaments are considered implicitly as parts of viscoelastic node-spring network.

The elastic propulsion theory predicts that squeezing of the ellipsoidal beads orients them so that motility along the long axes ensues, while geometric effect of spreading of branching actin filaments results in beads moving along their short axes. Separately, the existing theories cannot explain the observed bi-orientation of the beads. Our hybrid model posits that the combination of the elastic squeezing and geometric spreading leads to bi-orientation and reversible switching between two orientations, in agreement with the observations. To test the hybrid theory in the future, we propose to vary the bead geometry and concentrations of actin accessory proteins, thus modulating the network stiffness and interactions with the surface. Our model makes specific, nontrivial and testable predictions (see Figure 2, E–G) for such experiments.

The hybrid model reproduces the observed order of magnitude of curvatures of the trajectories in 2D and suggests that switching between the low- and high-curvature trajectories is caused by the temporary entrapment of the beads in the actin gel. The model predicts a Gaussian distribution of the curvatures for fast-moving beads due to random fluctuations of filament numbers and redistribution of actin around the bead's surface. In agreement with observations, our simulations show an additional sharp peak at zero curvature in the curvature distribution for slowly-moving beads. Importantly, the model suggests that elastic effects have little impact on the distribution of trajectory curvatures for fast-moving beads, while for beads that tend to be trapped in the actin cloud due to frequent filament attachments, the elastic effects are responsible for deviations from Gaussian distributions.

The hybrid model posits that the qualitative difference between two force-velocity measurements [18], [19] stems from the characteristic time difference: when the measurement is made over a long time interval [19], the viscoelastic recoil of the older, aging part of the network near the base of actin pedestal cancels protrusion and causes the concave-down force-velocity relation. On the other hand, when the force is clamped and the experiment is performed over shorter times [18], the concave-up force-velocity relation is predicted. A possible way to test our model is to use fluorescent speckle microscopy to measure the kymograph of material points of the actin network that move with the recoiling network away from the surface being pushed. We predict the resulting curves for two considered experiments in Figure S14 in Text S1. Note, that there are alternative explanations for the result [19]. For example, theory in [30] based on a representation of the actin network as a viscoelastic solid could predict a different kymograph. Finally, the model proposes that the shape of the surface does not qualitatively affect the shape of the force-velocity relation.

In the present form, our model has a number of limitations. The main one is that due to computational time limitations, we simulated the model in 2D as a simplification of a 3D system. So, rigorously speaking, all our results are applicable to cylindrical, rather than spherical objects. In Ref. [28], we already attempted the 3D modeling, albeit of an oversimplified model. Preliminary indications from that attempt are that most of the 2D model predictions survive in 3D. However, there are effects of higher dimension: 3D viscoelastic theory and experiment [27] suggest that ellipsoidal beads break through the actin cloud sideways, while [28] reports the observed lengthwise symmetry breaking of the ellipsoidal beads. This problem remains open, and thus more 3D modeling is necessary. In addition, helical and more complex trajectories of actin-propelled beads that have been observed in 3D environments [23], [24] cannot be captured by our 2D model. Furthermore, our model is coarse-grained and neglects important fine-scale processes such as hydrolysis of ATP bound to polymerized actin [31]–[33], exact actin branching angles [34], indirect synergy between capping and branching [35], molecular details of the nano-scale protrusion [36] and dependence of the branching rate on filament bending [37]. Future incorporation of these details into the model will clarify molecular nature of the mixture of nucleation-based and autocatalytic actin growth posited in the model.

Due to these limitations, our model does not capture some observed effects. Notably, the simulations do not reproduce observed hysteresis in the growth velocity of actin networks under force [19], which likely depends on complex dynamic features of the network [34], [38] that are not incorporated into our model. Similarly, not reproducing deviations from the Gaussian distribution of the curvatures of trajectories [25] likely means that some inhomogeneities in the distribution of actin nucleation promoting-factors not included into the model play an important role. These inhomogeneities and 3D effects also have to be built into the model to reproduce helical trajectories reported in [21], [23].

Another open question is relation of our model to other theories of the actin-based propulsion. Those include microscopic models of propulsion by tethered actin filaments [39], [40] that can in principle be used as boundary conditions for the viscoelastic actin gels and tested by simulations similar to those done here. Two mesoscopic models, very different from ours, were proposed recently. One of them considers excluded volume effects [41], another is a liquid of dendritic clusters model [42]; both of them successfully reproduce the concave-down force-velocity curve. It is likely that subtle physical effects on which these models are based complement elastic deformations and individual filament ratchet forces of our model. In the future, after including interactions of the filaments with cell membrane [43]–[46], contractile myosin effects [47] and more adequate actin rheology [48], our model can be applied to the general problem of cell protrusion.

## Materials and Methods

### Bead Motility Assays

Motility experiments on ellipsoidal beads were carried out in the lab of J. Theriot as previously described [28]. Briefly, 1-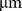 carboxylated polystyrene microspheres (Polysciences, Warrington, PA) were placed in a viscoelastic matrix (6% polyvinyl alcohol), heated to 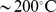, and stretched uniaxially. The film containing the beads was cooled and dissolved using an isopropanol/water mixture to recover the beads before functionalizing their surfaces with carboxylate. Electron microscopy showed that the beads had average dimensions of 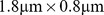, with an average aspect ratio of 2.2. His-tagged ActA was purified and adsorbed on the surface of beads at saturating amounts. ActA-coated beads were then added to *Xenopus laevis* egg cytoplasmic extract, which was diluted to 40% of the original protein concentration. The slide chamber depth was restricted using 2-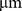 silica spherical beads. Note, that the ActA-coated motile beads were contained between two parallel coverslips and restricted from moving perpendicularly to the coverslips, and thus the trajectories of the beads were two-dimensional. All time-lapse sequences taken during the steady-state bead motility were acquired between 2 and 4 h after preparing the slide. Phase-contrast and fluorescence images were acquired as described in [28].

Spherical beads were prepared in the lab of J. Theriot as previously described [5], which is similar to that for ellipsoidal beads except for the stretching treatment. Bead trajectories were recorded at 10 s intervals.

For both experiments, positions and orientations of beads were computed from phase-contrast images and assembled into tracks as described in [28]. Smoothing of the instantaneous angular velocity values of the beads was generated using a weighted average of five nearest neighbors and a cubic equation as described in [28]. The angular velocity fit-in was generated using a seventh-order polynomial function. The curvature was obtained by dividing the resulting angular velocity by the instantaneous linear speed of the bead.

### Computational Model

In the hybrid model (Figure 1C), arrays of actin filaments interacting directly with the surface of the bead are treated as effective individual filaments, while other (not in touch with the surface) filaments are not modeled explicitly but rather treated as the network of elastic springs interconnected by nodes. The model is formulated and all simulations are done in 2D, which is a simplification of a 3D system. We assume that new filaments are created around the surface via a mixture of spontaneous nucleation, which has a spatially uniform rate along the bead surface, and autocatalytic branching processes, which has a rate proportional to the local density of existing filaments (not necessarily uniform in space). Separately, either of these processes produces a defective actin tail (see Figure S4 and discussion in Text S1). We also assume that newly created filaments immediately anchor to the network at their pointed ends which become new nodes of the network. In the simulations, this step is achieved by connecting each pointed end with undeformed springs to up to 4 neighboring nodes in the network that are within 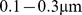 from the pointed end (see Figure S3 in Text S1). Thus, creation of new filaments dynamically expands the actin network. We treat filaments as elastic springs that are created in an attached and undeformed state. When stretched, attached filaments produce resisting forces that are proportional to their deformations. Attached filaments undergo detachment with a rate that increases exponentially with the load force. After detachment, filaments become free and are able to elongate and produce pushing forces against the obstacle. Free filaments are treated as linear elastic springs with the rest length growing with the polymerization rate. This rate is a function of the load on the barbed end of the filament; the function is given by the individual filament force-velocity relation that follows from the Brownian ratchet theory. The pushing force that a free filament exerts on the surface is computed as follows: at each time step, a virtual ‘penetration’ distance of the barbed end of the rest-length spring, corresponding to the filament, into the bead is computed. The filament is assumed to be deformed by this penetration distance, and respective elastic force is the pushing force. Free filaments can re-attach to the surface and get capped at constant rates.

Once capped, the filament is removed from the simulation, since in reality it will stop growing and cannot attach to the surface to exert pulling forces. However, the node corresponding to the pointed end of the filament remains, so this filament effectively becomes a part of the deformable network. We do not track the orientation of individual pushing filaments, but treat them as coarse-grained clusters of actual filaments that always push perpendicularly to the obstacle surface (see Figure 1D). As filaments exert forces on the obstacle, they also apply opposite forces to the elastic network that they are anchored to, causing network deformations (see Figure 1D). Similarly, the stress in the deformed network is transferred to the bead surface through the interacting filaments.

The deformation of the network is represented by the motion of nodes and springs in the network, which is obtained by moving all the nodes toward their force-equilibrium positions at each time step. For actin-propelled beads, we assume that the nodes in the network become immobile when they are more than a few microns away from the bead surface, representing the adhesion of the actin tail to the substrate. The bead moves and rotates to satisfy the force and torque balances from the filaments. For the force-velocity measurements, we fix the network at the bottom and allow all the rest nodes to move to reach force balance. The network undergoes disassembly, which is treated by removing the nodes and their connected springs from the network randomly with a rate proportional to the number of existing nodes. We have also included the effect of rupture of crosslinks by introducing a critical stretching force, above which the links break and get removed from the network. During the steady motion of beads, the creation and extinction rates of actin networks balance, causing a treadmilling actin tail behind the bead (Video S1). Effective viscoelastic behavior of the actin network emerges from the disassembly and breaking of the network. Further details about the model equations and parameters are described in Text S1.

## Supporting Information

Text S1 **Supplementary theoretical and computational methods.**
(PDF)

Video S1 **Simulation of an actin-propelled ellipsoidal bead with mesoscopic model, in which both the macroscopic elastic deformation of the tail and the microscopic branching of filaments are included.**
(MOV)

Video S2 **Simulation of an actin-propelled ellipsoidal bead with macroscopic elastic model alone, in which branching of individual filaments is not included.**
(MOV)

Video S3 **Simulation of an actin-propelled ellipsoidal bead with microscopic filament model alone, in which the elastic deformation of the tail is ignored.**
(MOV)

Video S4 **Simulation of an actin-propelled spherical bead with mesoscopic model, with all new filaments being created via autocatalytic branching.**
(MOV)

Video S5 **Similar to Video S4, except that all new filaments are created via spontaneous nucleation.**
(MOV)

Video S6 **Similar to Video S4, except that half of the new filaments being created via autocatalytic branching and the other half via spontaneous nucleation.**
(MOV)

Video S7 **Simulation of an actin-propelled spherical bead with mesoscopic model.**
(MOV)

Video S8
**Similar to Video S7, but with a lower detachment rate of** 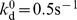 **.**
(MOV)

Video S9 **Simulated force-velocity measurement for actin pedestal pushing elastic cantilever.**
(MOV)

Video S10 **Simulated force-velocity measurement for a force-clamped actin tail growing from spherical bead.**
(MOV)
