# Supplementary material for: Mesoscopic Model of Actin-Based Propulsion
Source: PLoS Comput Biol. 2012 Nov 1;8(11):e1002764. doi: 10.1371/journal.pcbi.1002764 (PMC3486854; doi:10.1371/journal.pcbi.1002764)
Supplement: Text S1 — Supplementary theoretical and computational methods. (PDF) [file pcbi.1002764.s001.pdf]

Supporting Information for the manuscript 'Mesoscopic Model of Actin-Based Propulsion'

Jie Zhu[1], Alex Mogilner[1,*]
1 Department of Neurobiology, Physiology and Behavior and Department of Mathematics, University of California, Davis, CA 95616, USA
∗ E-mail: mogilner@math.ucdavis.edu

General description of the model

We model actin networks in a coarse-grained fashion similar to Ref. [1]. Near the bead surface, actin filaments are treated as individual Hookean springs with one end pinned to the rest of the network and the other interacting directly with the bead surface (see Figure S3). Away from the bead surface where actin filaments do not touch the bead surface, actin networks are coarse-grained into a 2D node-spring network with springs of the same stiffness. At the filament-network interface, individual filaments are anchored to the network with their minus-ends connected to nearby nodes of the network. We assume that these individual filaments generate forces according to the Brownian ratchet theory [2, 3]. As filaments exert forces on the bead, the same amount of forces will also be transferred back and deform the supporting network. The deformed network in turn applies stress to the bead surface through interacting filaments. Therefore, the distribution of actin forces along the bead surface is determined by the coupling between interacting filaments near the bead surface and the supporting networks away from the bead surface.

The dynamics of actin filaments are modeled as a stochastic process. Nascent filaments are generated at the bead surface via a mixture of spontaneous nucleation with a spatially uniform rate and autocatalytic branching process with a rate proportional to the local density of existing model filaments. Newly created filaments are assumed to be immediately incorporated into the existing network by turning their pointed ends to new nodes of the network (see Figure S3). This step is achieved by connecting each nascent pointed end with up to 4 neighboring nodes in the network with undeformed springs. We impose the following rules for the choice of neighboring nodes: each neighboring node is within a 0.05 to 0.8 μm distance from the nascent node to mimic the sub-micron mesh size of actual actin networks; to maintain a uniform network structure, no nodes are allowed to have more than 8 connections and the cosine of angle between each pair of connections is restricted to be less than 0.9; if more than 4 neighbors satisfy these rules, the nearest 4 from the nascent node are chosen. After connecting to 4 neighbors, each nascent node is allowed to be connected by other new nodes according to the same rules. Thus, creation of new filaments dynamically expands the actin network.

Free filaments can be capped, upon which filaments are removed from the simulation, since in reality they will stop growing and cannot attach to the surface to exert pulling forces. But the nodes corresponding to the pointed ends of the filaments remain. So, capped filaments effectively become part of the deformable network. Free filaments can also attach to the surface of the bead. Once attached, the filaments stop elongating and their barbed ends are associated with the surface at the contact position. When stretched, attached filaments produce resisting forces proportional to their deformations. Attached filaments undergo detachment with a rate that increases exponentially with the load force. After detachment, filaments become free again and are able to elongate and produce pushing forces against the obstacle.

Free filaments interacting directly with the bead are treated as Hookean springs that are created in an unattached and undeformed state. The rest lengths of those free filaments grow at the polymerization rate, which depends on the load on the barbed end of the filament. The load-dependent polymerization rate is given by the force-velocity relation of individual filaments that follows the Brownian ratchet theory. The pushing force that a free filament exerts on the surface of bead is generated by filament bending. Since the bending force is roughly proportional to the shortening of the projected length of the filament

along the undeformed axis, we approximate the pushing force to be the virtual 'penetration' distances of the barbed ends of the undeformed filaments into the bead. The penetration, of course, does not take place in reality. It is an approximation of the bending effect of actual pushing filaments which presses against bead's surface and generate elastic forces. Respective spring constant of the filaments is assumed to be the same as that of the network springs. Since the average orientation of force-generating filaments is locally normal to the bead surface, we assume the orientation of model filaments to be perpendicular to the local bead surface. Therefore, the angular spring element at the filament-node connection is ignored and filaments are allowed to pivot about their pointed ends to maintain the normal direction.

Filaments are assumed to be anchored to the node-spring network at the back. The deformation of the network is represented by the motion of nodes and springs in the network, which is obtained by moving all the nodes toward their force-equilibrium positions at each time step. For actin-propelled beads, we assume that the nodes in the network become immobile when they are more than 1 μm away from the nearest point on the bead surface, representing the adhesion of the actin tail to the substrate. The bead moves and rotates so that both the total force and the total torque from the model filaments to the bead are zero. The stress in the network influences the motion of the bead in multiple ways. Since the model filaments are small springs between the elastic gel and the bead, the resulting force on the bead comes from the nontrivial force balance between the gel and the filaments. The network stress also affects the local growth rate of embedded filaments via the force-velocity relation, modifying the local property of filament springs and thus the force on the bead. If the deformation of the network is ignored, filaments are effectively embedded onto a rigid surface and are mutually independent, which is equivalent to the ratchet model. On the other hand, if the detailed microscopic filament dynamics such as the local force-velocity relation is ignored and the stress is assumed to be uniform along the bead surface, such boundary condition makes our model equivalent to the elastic propulsion model.

Although there is no angular springs connecting the nodes in the network and the elastic springs can pivot freely about the nodes, the network can still sustain shear, compression and stretch. The model gives rise to the bulk elasticity of the actin gel because on average there are 4 links per node so that the links-to-nodes ratio is 4. Studies (e.g. Ref. [4]) have shown that a network is able to sustain shear, compression and stretch if the links-to-nodes ratio is greater than the spacial dimension. For our 2D network, the links-to-nodes ratio is greater than 2. Therefore, our network is rigid and can sustain shear even without angular stiffness to the springs.

For the force-velocity measurements, we fix the network nodes at the bottom to the surface and allow the rest of the nodes to move to reach the force balance. The network undergoes disassembly, which is treated by randomly removing nodes and their connected springs from the network at a rate proportional to the number of existing nodes. We have also included the effect of rupture of crosslinks by introducing a critical stretching force, above which the links break and get removed from the network. On time scales of the order of a few tens of seconds (determined by the slow disassembly rate constant of about 0.01 s^{-1}), the network is elastic and can withstand compression, shear and stretch. On longer time scales, the network disassembles to a significant extent and starts flowing slowly, which resembles the viscoelastic behavior of Maxwell materials.

Simulation Procedures

At the beginning of the simulation, a thin layer of spring-node network is created around the bead (or below the cantilever) as the initial foundation of the network. The nodes in the network are distributed randomly in the region with a density k_n/lv_b^*, where k_n is the total nucleation rate of filaments, l is the length of the bead-network interface and v_b^* is the estimated speed of bead. The initial connection of springs in the network is obtained using a Delaunay triangulation algorithm. All springs are created in an undeformed state.

The simulation time step is chosen to be $\Delta t = 0.1$ s. At each time step, a total number of $[k_n \Delta t]$ new

nodes are created along the surface of the bead (the fractional part, $k_n\Delta t - [k_n\Delta t]$, will be carried over to the next time step), representing the pointed ends of nascent filaments. Among them, half are assumed to be created via autocatalytic branching, the rate of which is proportional to the local density of existing network nodes; the other half are assumed to be created via spontaneous nucleation, the rate of which is independent of the density of existing filaments but is uniform along the surface of the load. To include the "brushing" effect for actin-propelled beads, we further bias the spontaneous nucleation toward the back of the bead surface such that the front-to-back ratio is 1:2. Once created, each node is immediately connected to up to 4 neighboring nodes by undeformed elastic springs, representing the anchoring of the filaments into the network. For a network of N nodes, a total of $[k_{dis}N\Delta t]$ randomly selected nodes, together with their connected springs, are removed from the simulations in each Δt, representing the disassembly of the network (similarly, the fractional part will be carried over to the next time step). We have also included the rupture of networks in the model. Once a network spring is stretched beyond a 15 pN force, it is removed from the simulation.

The dynamics of filaments is considered as a Poisson process. If a free filament remains in contact with the bead surface for an average time of $1/k_a$ where k_a is the attachment rate constant, it converts to an attached filament, the barbed end of which binds to the surface at the contact point. If a free filament remains unattached for an average time of $1/k_c$ where k_c is the capping rate constant, it is considered to be capped. Attached filaments cannot elongate nor can be capped, but can exert pulling forces on the bead surface. The pulling force from each attached filament is determined by the relative displacement from the attachment point to the position of the tip if the filament is undeformed. The time that a filament remains attached is calculated as follows: at each time step, the probability that the filament will detach increases by $k_d\Delta t$, where k_d is the detachment rate depending on the current load of the filament. Once the probability reaches 1, the filament is considered to detach and become free.

We do not track the orientation of individual pushing filaments, but treat them as coarse-grained clusters of actual filaments that always push perpendicularly to the obstacle surface. The lengths and loading forces of filaments are computed self-consistently as follows: at each time step, the equilibrium position of each filament is calculated by balancing the elastic forces from both the bead's surface and the node-spring network that the filament is attached to. To ensure the entire network reaches force balance, the above procedure is repeated multiple times until the variation in total elastic energy is less than 1% of the average value. Then, the growth rate for each filament is computed from the force-velocity relation and the filament length is updated accordingly. At the next time step, new position of each filament is found, and all the above steps repeat. Simultaneously, positions of the bead and all nodes are updated. To speed up the relaxation time for the network to reach force balance in the cantilever experiments, we further divide the entire network into 10 segments of equal length along its length and impose the condition that the total force on filaments across each interface is equal to the external force on the cantilever. The simulation code is written in C and the graphics is rendered using OpenGL. Simulations are performed on a cluster of 64 Xeon 2.4 GHz CPU cores. The typical simulation time for each individual simulation is a few hours per CPU core.

Choice of Parameters

Our 2D network represents a slice of the 3D network. Due to computer time limitations, we simulate only a small fraction of the network with about 100 interacting filaments at the network's boundary. From Eqs. 5 and 7 below, we estimate that choosing $k_n/k_c = 20\ \mu\text{m}^{-1}$ leads to about 60 free filaments around a $R = 1\ \mu$m bead. For tractable numerical simulations, we choose $k_n = 2\ \mu\text{m}^{-1}\text{s}^{-1}$ and $k_c = 0.1\ \text{s}^{-1}$. We find that the numerical results are insensitive to the actual values of k_n and k_c as long as their ratio is fixed. There is no direct measurement of k_a, so we choose the rate constant $k_a = 1\ \text{s}^{-1}$ characteristic for molecular association rates in the cell. We find that our simulation results are insensitive to the actual value of k_a, but the ratio of k_a to k_d^0 is important. Our results show that micron-sized spherical

beads often get trapped into the actin gel for $k_d^0 < k_a$ but can have consistent motion at $k_d^0 > k_a$. The simulations also show that ellipsoidal beads obtain orientational bi-stability, so that the probabilities to move lengthwise and sideways are the same, at $k_d^0 \approx 2.7k_a$, therefore, we choose $k_d^0 = 2.7$ s^{-1}.

The value of the linear spring constant k_s of springs in the 2D network is estimated from the Young's modulus (Y) of a 3D network. We assume that our 2D network has a similar mesh size (ξ) to that in the 3D network. Since the compression modulus of the 2D node-spring network has the same order of magnitude as the spring constant of individual springs in the network [5], we estimate $k_s \approx \xi Y$. The value of ξ in the simulations can be obtained from k_n and v_b. Since actin-propelled spherical beads in our 2D simulations have characteristic speed $v_b \approx 0.03$ μm/s and $k_n = 2$ μm^{-1}s^{-1}, each second there will be an average of 2 nodes created in a 1 μm $\times$ 0.03 μm domain near the bead surface. Then, the average node-node distance (mesh size) is $\xi \approx \sqrt{0.03/2} \approx 0.1$ μm. Therefore, to represent a 3D network with $Y = 3$ kPa, we choose $k_s = \xi Y \approx 300$ pN/μm in our 2D node-spring network.

The value of $v_0 = 50$ nm/s is estimated as an upper limit of the observed speed of $v_b \approx 30$ nm/s for micron-sized spherical beads. These beads typically form actin tails of a few microns long. For a tail of $l = 4$ μm long, the network disassembly rate is estimated as $k_{dis} \approx v_b/l \approx 8 \times 10^{-3}$ s^{-1}. It corresponds to a characteristic life time of about 100 s of the actin network.

Autocatalytic Branching versus Spontaneous Nucleation

There are two possible mechanisms for the nucleation of new filaments: autocatalytic branching and spontaneous nucleation. In the simulation, we keep the global nucleation rate, which is assumed to be proportional to the size of the bead, a constant. We have tested these two mechanisms individually with simulations (see Figure S4, A and B), and found that each mechanism alone does not produce the observed motion of the bead. With just autocatalytic branching, simulations show that filaments quickly concentrate into a narrow tail behind the moving bead (Video S4), which is different from experimental observations. The reason is that new filaments are more likely to be created near the region with denser existing filaments, causing a positive feedback for the accumulation of filaments into a narrow tail. With just spontaneous nucleation, new filaments are created uniformly around the bead surface. Simulations show that an initially asymmetric actin cloud quickly develops into a uniform 'cocoon' around the bead, stopping the bead's motion (Video S5). To produce the consistent motion of bead with the width of the tail being comparable to the bead's size, we combine the autocatalytic branching and spontaneous nucleation of filaments. By choosing a global branching rate density of $k_n/2$ and a total spontaneous nucleation rate density of $k_n/2$, we find that the bead can move consistently with a wide tail similar to the observations (Video S6, Figure S4C). The branching process is modeled by generating nascent model filaments with a local rate proportional to the density of nearby (averaged within a window of 0.1 micron width) filaments. Note that we are not positing, of course, that the nucleation is Arp2/3 independent; the combination of the nucleation and branching is simply a convenient modeling way to capture both spreading tendency of the growing actin network and autocatalytic character of this growth.

For the actin tail, a low-density core has been reported [6]. Our model, actually, predicts this effect for a simple geometric reason: as the bead is propelled forward, all actin growing on the side of the bead ends up in the margin of the actin tail, while actin growing at the rear end of the bead is left in the core of the tail. Because the side surface of the bead has a much smaller projected area onto the cross section of the tail than the rear surface does, a similar density of actin around the bead will cause a higher density of actin in the margin of the tail than in the core. We did not investigate the mechanical consequences of that effect: it could in principle stiffen the tail to some extent because effectively it increases the density of the tail; it is also likely that in the experimental system the effect is partially due to the increased alignment in filaments in the tail margins [6] comparing to those in the core, which is beyond the scope of our model.

Our model also does not include the recently discovered increased branching rate in highly bent

filaments [7]. Qualitatively, this effect means that the local actin production rate is a growing function of the local stress. According to our calculations, the stress is highest near the edge of the tail-bead interface. Increasing the actin density there will lead to increased local force on the sides of the bead, which is supposed to bias the bead to move along its long axis. Furthermore, the increased actin density probably also causes the stiffening of the actin network near the margin of the tail, which is likely to increase the biased motion of the bead along its long axis. However, the spatial resolution of our simulations and model geometry are too crude for careful investigation of this effect. The impact of this important phenomenon on the actin-based propulsion is left for future investigation.

Orientation of Actin Propelled Ellipsoidal Beads

The beads in the experiment [8] were confined in a chamber of only 2 μm height, which is comparable to the size of the bead. Therefore, both the bead's motion and the actin network can be regarded as 2D. In the following simulations of our 2D mesoscopic model, each data point is obtained from at least 100 individual simulations with each being 10^4 s long. Therefore, each data point is averaged over 10^6 s and the error is expected to be within 10%.

We define the bead's orientation angle θ to be the angle between the bead's long-axis and the axis of its immediate tail. The simulated $v_{\rm b}$-θ relation is shown in Figure S5. We find that beads moving along their long-axes are slightly slower than those moving along their short-axes. Beads moving along their long-axes have a broader distribution of speeds, most of which range from 10 nm/s to 30 nm/s. Beads moving along their short-axes have a much smaller spread of speeds, which range from 25 nm/s to 35 nm/s. The orientation of an ellipsoidal bead with respect to its tail is determined by both the torque-induced rotation of the bead and the motion-induced reorientation of the tail around the bead. Below, we develop a semi-analytical continuous model that gives qualitative insight to the problem and allows better understanding of the numerical simulation results reported in the main text.

Torque-induced rotation of the bead

To find the torque applied to the bead by its tail, we first ignore the macroscopic elastic deformation of the tail and calculate the microscopic forces exerted by individual actin filaments. Then, we consider a concentrated force on the side of the bead to balance the sideways forces from all the individual filaments, representing the macroscopic elastic reaction of the tail. The total torque on the bead is then obtained from these two forces.

According to the tethered ratchet model [3], the elongation of free filaments generates pushing forces and the stretching of attached filaments produces pulling forces. Thus, we consider two populations of actin filaments at the microscopic level, free and attached. We treat the barbed-end density of free filaments ($\rho_{\rm f}$) and attached filaments ($\rho_{\rm a}$) as continuous functions along the bead surface. In our calculations, we consider the dynamics of filaments including branching, attachment, detachment and capping: new filaments branch off from existing filaments near the bead surface; the barbed ends of filaments can either attach to or detach from the bead surface; filaments switch between these two states with certain rates; the barbed ends of free filaments can be capped by capping proteins and thus stop their dynamics. In this section, we treat the actin tail as an infinitely rigid network. Since any part of the rigid tail that is in front of the bead will stop the bead's motion, we further assume that filaments exist only at the back of the bead (the bead-tail boundary, $\rm P_1$ and $\rm P_2$, is where the surface tangents are parallel to $\vec{v}_{\rm b}$, see Figure S6A). This assumption is consistent with the observed lower density of actin networks in front of the moving beads. All kinetic processes described above determine the actin (densities $\rho_{\rm a}$ and

ρ_f) dynamics governed by the equations:

$$\frac{\partial \rho_\mathrm{a}}{\partial t} = k_\mathrm{n} - k_\mathrm{d}\rho_\mathrm{a} + k_\mathrm{a}\rho_\mathrm{f} \tag{1}$$

$$\frac{\partial \rho_\mathrm{f}}{\partial t} = k_\mathrm{d}\rho_\mathrm{a} - (k_\mathrm{a} + k_\mathrm{c})\rho_\mathrm{f}, \tag{2}$$

where k_n, k_a, k_d and k_c are the average rate constants for nucleation (per unit length along the bead surface), attachment, detachment and capping of filaments, respectively. The estimated values of k_n, k_a and k_c are based on previous work [3,9] and are listed in Table S1. The value of k_d, however, depends on the relative motion between the attached filament and the bead surface. We approximate the previous calculation [3] of k_d as follows:

$$k_\mathrm{d} = \frac{k_\mathrm{d}^0}{\int_0^\infty x \exp[ux + (1 - e^{ux})/u]\,\mathrm{d}x} \approx k_\mathrm{d}^0\left(1 + \frac{v_\mathrm{b}}{2v_\mathrm{c}}\right), \tag{3}$$

where $k_\mathrm{d}^0 = 2.7\ \mathrm{s}^{-1}$ is the zero-force detachment rate and $u = v_\mathrm{b}/v_\mathrm{c}$ is the scaled relative velocity. Here, $v_\mathrm{c} = f_\mathrm{b}k_\mathrm{d}^0/k_\mathrm{s} \approx 30$ nm/s is a velocity scale with $k_\mathrm{s} = 300$ pN/μm being the spring constant of the attachment bond and $f_\mathrm{b} \approx 10$ pN being the strength of the bond [3]. For the typical value of $v_\mathrm{b} \approx 30$ μm/s, we have $k_\mathrm{d} \approx 1.5k_\mathrm{d}^0$. This approximation introduces an error less than 10% for $v_\mathrm{b} < 5v_\mathrm{c} = 150$ nm/s.

At the steady state, Eqs. 1 and 2 give:

$$\rho_\mathrm{a} = \frac{k_\mathrm{a} + k_\mathrm{c}}{k_\mathrm{d}}\frac{k_\mathrm{n}}{k_\mathrm{c}}, \tag{4}$$

$$\rho_\mathrm{f} = \frac{k_\mathrm{n}}{k_\mathrm{c}}. \tag{5}$$

Therefore, the number of attached (N_a) and free (N_f) filaments are:

$$N_\mathrm{a} = \frac{s_0}{2}\rho_\mathrm{a}, \tag{6}$$

$$N_\mathrm{f} = \frac{s_0}{2}\rho_\mathrm{f}, \tag{7}$$

where $s_0 \approx 4.8$ μm is the perimeter of the ellipse and the factor 1/2 is from the assumption of filaments nucleation being restricted to half of the surface. The total number of filaments is

$$N = N_\mathrm{a} + N_\mathrm{f} = \frac{k_\mathrm{a} + k_\mathrm{c} + k_\mathrm{d}}{k_\mathrm{d}}\frac{k_\mathrm{n}}{k_\mathrm{c}}\frac{s_0}{2}. \tag{8}$$

We checked by numerical simulations that $N_\mathrm{a}/N_\mathrm{f} \approx k_\mathrm{a}/k_\mathrm{d}^0$ in a wide range of parameters (the fitted slope of the straight line to the $N_\mathrm{a}/N_\mathrm{f}$ vs $k_\mathrm{a}/k_\mathrm{d}^0$ relation is ≈ 0.94). Thus, in the simulations we change $N_\mathrm{a}/N_\mathrm{f}$ by varying the value of k_a.

The viscous drag on the bead is negligible compared to the actin propulsion forces. To calculate the actin propulsion force on the bead, we define the bead's geometry in its own frame-of-reference as $x^2/a^2 + y^2/b^2 = 1$, where A=0.5 μm and b=1 μm are the short- and long-axes of the bead, respectively; we also define a one-dimensional (1D) curvilinear coordinate s with origin at $x = 0$ and $y = b$ in the bead frame (see Figure S6A) and the positive direction being clockwise along the bead surface. Let $\hat{n}_s$ be the unit outward normal vector of the bead surface at s, its value at point (x, y) on the bead surface is $\hat{n}_s = (x^2/a^4 + y^2/b^4)^{-1/2}(x/a^2, y/b^2)^\mathrm{T}$. We assume that free filaments push perpendicularly against the bead surface following the prediction of the Brownian ratchet theory [2]:

$$\vec{f}_\mathrm{f} = f_0 \ln(v_0/v_\perp)(-\hat{n}_s), \tag{9}$$

where $f_0 \approx 1.5$ pN is the force scale, $v_0 = 50$ nm/s is filaments' zero-load polymerization speed, and $v_\perp = |\vec{v}_\mathrm{b} \cdot \hat{n}_s|$ is the relative velocity between the bead and filament in the normal direction. The pulling force from an attached filament can be estimated [3] as

$$\vec{f}_\mathrm{a} \approx -k_\mathrm{s}\vec{v}_\mathrm{b}/k_\mathrm{d}. \tag{10}$$

Then, the total force from all the filaments is

$$\vec{F}_\mathrm{fil} = \int_0^{s_0} (\rho_\mathrm{f}\vec{f}_\mathrm{f} + \rho_\mathrm{a}\vec{f}_\mathrm{a})\,\mathrm{d}s. \tag{11}$$

Because of the different directions of pushing and pushing forces (see Figure S6B), the resulting $\vec{F}_\mathrm{fil}$ always has a non-zero component perpendicular to the direction of $\vec{v}_\mathrm{b}$, if $\vec{v}_\mathrm{b}$ is not parallel to any axes of the bead. This force component will push the bead sideways, causing an additional opposing force from the tail in that direction. Defining the tail's frame-of-reference X-Y such that the Y-direction is always parallel to $\vec{v}_\mathrm{b}$ (see Figure S6), this opposing force can be expressed as

$$\vec{f}_\mathrm{opp} = -\big(\vec{F}_\mathrm{fil} \cdot \hat{e}_X\big)\hat{e}_X, \tag{12}$$

where $\hat{e}_X$ is the unit vector along the X-direction. For reasons of simplicity, we assume that $\vec{f}_\mathrm{opp}$ is caused by free filaments that are concentrated at the opposing side of the bead-tail boundary (P_1 in Figure S6A).

We define the positive direction of torque to be the direction of increasing θ (clockwise for Figure S6A). From the forces calculated above, the total torque on the bead can be obtained as

$$\vec{T} = -\int_0^{s_0} \vec{r}_s \times [\rho_\mathrm{f}\vec{f}_\mathrm{f} + \rho_\mathrm{a}\vec{f}_\mathrm{a} + \vec{f}_\mathrm{opp}\delta(s - s')]\,\mathrm{d}s, \tag{13}$$

where $\vec{r}_s$ is the displacement from the bead center to a point on the bead surface at s (see Figure S6A), δ is the Dirac delta function, and s' is the location of $\vec{f}_\mathrm{opp}$ in the s-coordinate. The torque-induced angular velocity of the bead is

$$\omega_1 = \mu_\mathrm{r}\vec{T} \cdot \hat{e}_z, \tag{14}$$

where μ_r is the rotational mobility of the bead and $\hat{e}_z = \hat{e}_x \times \hat{e}_y$ is a unit vector in the z-direction. To estimate μ_r, we first consider a spherical bead with a comparable radius $\sqrt{ab}$. As the bead rotates at angular velocity ω, N_a attached filaments will pull the bead along the tangent of the surface to resist the motion with an average force per filament $f_\mathrm{a} \approx k_\mathrm{s}\omega R_0/k_\mathrm{d}$ (see Eq. 10). The torque generated by these filaments is $T_0 = N_\mathrm{a} f_\mathrm{a}\sqrt{ab}$. Then, the rotational mobility of this spherical bead is $\mu_{\mathrm{r},0} = \omega/T_0 \approx k_\mathrm{d}/N_\mathrm{a}k_\mathrm{s}ab \approx 1.2 \times 10^{-4}$ $\mathrm{pN}^{-1}\mu\mathrm{m}^{-1}\mathrm{s}^{-1}$. For an ellipsoidal bead, the resisting force from the filaments will have both tangential and normal components, due to the asymmetric shape. The normal components of the resisting forces will slow down the rotation further. We thus introduce a correction factor $0 < c < 1$ to obtain the rotational mobility of ellipsoidal beads: $\mu_\mathrm{r} = c\mu_{\mathrm{r},0} \approx ck_\mathrm{d}/N_\mathrm{a}k_\mathrm{s}ab$. We treat c as a fitting parameter to get the bi-stability of the orientation of the ellipsoidal beads (see below). Generally, one would expect that a stiffer network will have a higher c because it generates opposing normal forces more effectively.

By numerically solving Eqs. 9–14, we find that ω_1 is always negative for $0 < \theta < 90°$ (Figure S6D). This indicates that the bead always tends to move along its long-axis.

Actin-remodeling-induced turning of tail around the bead

In addition to the torque-induced rotation of the bead, the reorientation of the tail around the bead surface can also lead to relative rotation between the bead and its tail. This reorientation of the tail

is caused by the change of the bead's direction of motion, which, in turn, is a result of change in the direction of pushing force.

To understand this effect quantitatively, we consider a bead with initial direction of motion being close to its long-axis. In the bead's frame-of-reference, we define points P_1 and P_2 to be the left and right boundaries of the tail on the bead surface, respectively, and θ to be the angle between line P_1P_2 and the short-axis of the bead (x-axis in Figure S7A). Angle θ describes the direction from which the tail pushes the bead. We assume that the pushing filaments generate a uniform pressure on the rear half of bead between points P_1 and P_2. The torque generated by this pressure is zero due to symmetry. Therefore, in the lab frame, the bead will not rotate about its center but simply move along the direction of the pushing force. Because the pushing pressure is uniform, the force components that are parallel to line P_1P_2 cancel out, which leaves the pushing force perpendicular to line P_1P_2. Thus, the bead tends to move in the direction perpendicular to line P_1P_2 ($\vec{v}_\mathrm{b}$ in Figure S7A). As the bead moves along, the actin tail keeps remodeling itself, so that the tail always interacts with the bead from behind. The bead-tail boundary will then shift gradually from P_1P_2 to $P'_1P'_2$, where the tangents of the bead surface are parallel to $\vec{v}_\mathrm{b}$ (see Figure S7B). The angle between line $P'_1P'_2$ and the x-axis, θ', can be found as follows. Since the bead's direction of motion is $\vec{v}_\mathrm{b} = v_\mathrm{b}(\sin\theta, \cos\theta)^\mathrm{T}$, the normal to the bead surface at P'_1 is $\hat{n}'_1 \propto (x'_1/a^2, y'_1/b^2)^\mathrm{T}$, where x'_1 and y'_1 are the x- and y-coordinates of point P'_1, respectively. Since $\vec{v}_\mathrm{b} \perp \hat{n}'_1$, we have $\vec{v}_\mathrm{b} \cdot \hat{n}'_1 = 0$, which gives $x'_1 \sin\theta/a^2 + y'_1 \cos\theta/b^2 = 0$. We get

$$\theta' = \tan^{-1}\left|\frac{y'_1}{x'_1}\right| = \tan^{-1}\left(\frac{b^2}{a^2}\tan\theta\right). \tag{15}$$

Because $\theta' > \theta$ for all $0 < \theta < 90°$, the bead-tail boundary will keep tilting until the bead is moving along its short-axis. Although the bead does not rotate in the lab frame, the direction from which the tail interact with the bead changes continuously, causing relative rotation between the bead and its tail. If the bead moves close to its short axis, one finds opposite results: $\theta' < \theta$ for all $0 < \theta < 90°$ (see Figure S7, C and D), consistent with the result that the bead prefers moving along its short-axis.

The angular velocity of this rotation can be obtained by estimating the time required for remodeling the actin tail. Stability analysis of Eqs. 1 and 2 shows that the characteristic turn-over rate for actin is

$$k_0 = \frac{1}{2}\left[(k_\mathrm{a} + k_\mathrm{c} + k_\mathrm{d}) - \sqrt{(k_\mathrm{a} + k_\mathrm{c} + k_\mathrm{d})^2 - 4k_\mathrm{c}k_\mathrm{d}}\right] \approx \frac{k_\mathrm{c}k_\mathrm{d}}{k_\mathrm{a} + k_\mathrm{c} + k_\mathrm{d}}. \tag{16}$$

The approximate relation holds for $(k_\mathrm{a} + k_\mathrm{c} + k_\mathrm{d})^2 \gg 4k_\mathrm{c}k_\mathrm{d}$. We estimate the turn-over time of actin to be twice the inverse of k_0:

$$\tau_0 \approx \frac{2}{k_0}. \tag{17}$$

For the parameter values listed in Table S1 and $k_\mathrm{d} \approx 1.5k_\mathrm{d}^0$, we find $\tau_0 \approx 25$ s.

Then, the angular velocity of the bead with respect to its tail can be estimated as

$$\omega_2 = \frac{\mathrm{d}\theta}{\mathrm{d}t} \approx \frac{\theta' - \theta}{\tau_0} \approx \frac{k_\mathrm{c}k_\mathrm{d}}{2(k_\mathrm{a} + k_\mathrm{c} + k_\mathrm{d})}\left[\tan^{-1}\left(\frac{b^2}{a^2}\tan\theta\right) - \theta\right]. \tag{18}$$

The result is shown in Figure S7E. Since $\omega_2 > 0$ for $0 < \theta < 90°$, θ will keep increasing until it reaches $90°$, which corresponds to the bead's moving along its short-axis.

We have also performed stochastic simulations to study the actin-remodeling-induced rotation of tail. In the simulations, we let individual filaments nucleate at the rear half of the bead and push against it. We treat filaments as elastic springs and explicitly track the dynamics and deformation of each individual filament. To eliminate the effect of torque-induced rotation, we do not allow the bead to rotate in the lab frame, but allow it to move according to the direction of the total spring force exerted on it. We find that even if the orientation of the bead in the lab frame is fixed, the bead always tends to change its

direction of motion such that it moves along its short-axis. We plot the relative angular velocity of the bead with respect to its tail as a function of the angle (Figure S7E), and find good agreement with our analytical prediction.

Bead's overall rotation relative to its tail

The bead rotation with respect to its tail is determined by both the torque from the tail and the motion-induced reorientation of the tail. The torque-induced rotation describes the rotation of the bead when the orientation of the tail is fixed in the lab frame, while the motion-induced reorientation of the tail describes the turning of the tail when the orientation of the bead is fixed in the lab frame. The overall angular velocity of the bead with respect to its tail can be expressed as the sum of Eqs. 14 and 18:

$$\omega \approx \omega_1 + \omega_2. \tag{19}$$

Experiments show that half of the beads moving along their long-axes and the other half moving along their short-axes. This indicates that $\theta = 0$ and $90°$ are the stable points for rotations. We hypothesize that ω should have a sigmoidal shape: $\omega < 0$ for $0 < \theta < 45°$ and $\omega > 0$ for $45° < \theta < 90°$. We find that choosing $c = 0.53$ gives such a shape (Figure S8). This indicates that if a bead initially moves in a direction close to its long-/short-axis, it will keep moving along its long-/short-axis. The critical orientation at which beads have approximately equal chances to take either orientation is about $45°$.

For a lower value of parameter c, i.e. $c = 0.4$, which represents a lower rotational mobility and thus a stiffer network, ω becomes mostly positive. Then, the bead tends to rotate to $\theta = 90°$. This result is consistent with our simulation of the mesoscopic model (see Figure 2F in the main text): beads propelled by a stiff network tend to move along its short-axis. For higher values of c, our calculations show that the fraction of negative ω always increases with c, indicating increased probability of motion along the long-axis. This contradicts our simulation results of the mesoscopic model (see Figure 2F in the main text). Our explanation is that free filaments push less efficiently when they are anchored to a softer network. Since this effect is not included in the above calculations, our analytical model probably works best in the regime of high network rigidity.

The aspect-ratio dependence of beads' orientation (see Figure 2 in the main text) can be explained by the nonlinear dependence of beads' angular speed on the aspect ratio (see Eqs. 13, 14 and 18). Note that the aspect ratio has two contributions to ω_1: one is through s_0 and the other is through term $\vec{r}_s$, both of which depend nonlinearly on the aspect ratio. Numerical calculation shows that both ω_1 and ω_2 increases with the aspect ratio. But ω_1 has a concave-up shape and ω_2 has a concave-down shape. As a result, ω_2 increases more rapidly with the aspect ratio than ω_1 does if the aspect ratio is close to 1, leading to an increased chance for the beads to move along their short axes. For aspect ratio greater than 2, the trend is the opposite, causing an increased chance for the beads to move along their long axes. Since spherical beads do not have any preference on orientations, a peak of probability for beads moving along their short axes can be formed between aspect ratios 1 and 2.

We have also studied how the network's stiffness affects the orientation of beads of different aspect ratios (see Figure S9). For networks of intermediate stiffness (Y = 1–8 kPa), the probability for beads to move along their long axes increases significantly from about 20% to 80% as the aspect ratio increases from 1.5 to 2.5. But for softer or stiffer networks, the probabilities of orientations of beads are insensitive to beads' aspect ratio. Our explanation is that only at intermediate stiffness the elastic forces matter. When this is the case, making the bead more elongated makes the long axis preferred, as shown in the main text.

For the effect of filament attachments on the orientation of beads, both free and attached filaments produce torques to align the beads along their long axes (see Figure S6, C and D), while the actin-remodeling-induced turning always aligns the beads along their short axes (see Figure S7E). Increasing the ratio of $N_\mathrm{a}/N_\mathrm{f}$ while keeping $N_\mathrm{a} + N_\mathrm{f}$ constant raises the fraction of torque from attached filaments,

the magnitude of which increases more rapidly with θ near $\theta = 0$ (see Figure S6C), biasing the extreme value of ω_1 toward $\theta = 0$ (see Figure S6D). On the other hand, ω_2 is not affected by N_a/N_f. The combined effect is that the range of θ with a negative ω increases, leading to a higher chance for beads to move along their long axes. Finally, note that if we integrate the angular velocity of the beads over the orientation angle, the integral has the form of a double-well potential, which can be considered as an energy landscape for the over-damped movement of the beads. Adding effective angular diffusion could lead to the double-peaked probability distribution. This argument was also used in [8] to estimate the frequency of beads' turning.

Our numerical experiments described below indicate that the network is elastic up to a time scale of a few tens of seconds. Only on longer time scales does the network flow. The slowest bead's movement we deal with take place with rate $\sim$ 30 nm/s. In about 100 s, the bead is propelled by a few microns, so the weak and flowing part of the network is a few bead lengths away from the bead surface. Stresses and strains so far away from the bead are irrelevant for the bead propulsion, as previous experimental and theoretical studies demonstrated. The crucial layer of actin gel is the closest layer, the thickness of which is similar to the bead's size. In this layer, the network is created within $\sim$ 30 s and is elastic.

Trajectory of Actin-Propelled Spherical Beads

In the simulations, each data point was obtained from 200 individual simulations with each being 3600 s long. Therefore, the error is expected to be less than 10%. The average speed of simulated bead was about 0.03 μm/s. To obtain the curvature distribution of the trajectory $P(\kappa)$, we first smooth the simulated bead's trajectory with a Gaussian smoothing function. We choose a smoothing window size of about 1 μm to remove the high frequency noises. We then calculate the local curvature of the trajectory from the 2 neighbors that are 2.5 μm from each side of that location. These window sizes are chosen to be comparable to the observed curvature of about 0.1 μm^{-1} [10, 11].

In the main text, we derived the equation:

$$\frac{1}{\kappa_{\text{rms}}} \approx \frac{4}{\pi}\sqrt{N} v_{\text{b}} \tau_0. \tag{20}$$

The typical time τ_0 over which the directional bias persists is the turn-over time of the actin network, which we estimate to be $\tau_0 \approx 2(k_{\text{a}} + k_{\text{c}} + k_{\text{d}})/k_{\text{c}} k_{\text{d}} \approx 25$ s for $k_{\text{a}} \sim k_{\text{d}} \gg k_{\text{c}}$ and the parameter values listed in Table S1. Here, we estimate $k_{\text{d}} \approx 1.5 k_{\text{d}}^0$. Then, the typical angular velocity of the turning is $\omega_{\text{rms}} \approx \Delta\theta/\tau_0$, and the root-mean-square value of the curvature is $\kappa_{\text{rms}} = \omega_{\text{rms}}/v_{\text{b}} \approx \pi/4\sqrt{N} v_{\text{b}} \tau_0$.

To check whether our simulation results agree with the above relation, we obtain the values of κ_{rms}, N and v_{b} directly from simulations. From Eq. 17 and $k_{\text{d}} = (k_{\text{a}} + k_{\text{c}}) N_{\text{f}}/N_{\text{a}}$ (see Eqs. 4–7), τ_0 is estimated to be

$$\tau_0 \approx \frac{2N}{k_{\text{c}} N_{\text{f}}}, \tag{21}$$

where N_{f} is also obtained directly from simulations. We have varied k_{a}, k_{c}, k_{d}^0, k_{n}, k_{s}, R and v_0 individually to see if Eq. 20 holds (see Figure 4E in the main text). The simulated results of how N, v_{b}, τ_0 and κ_{rms} depend on these parameters are shown in Figures S10 and S11. Since changing any one of k_{a}, k_{c}, k_{d}^0, k_{n}, k_{s}, R and v_0 potentially changes the values of N, v_{b} and τ_0, it is challenging to obtain an explicit expression of how κ_{rms} depends on these individual parameters. Nevertheless, our simulation results provide a qualitative understanding of how individual parameters affect κ_{rms}.

i) Increasing k_{a} leads to a linear increase in both N (see Eq. 8) and τ_0 (see Eq. 21), but causes a decrease in v_{b} due to increased number of attached filaments (see Eq. 6). The total effect on κ_{rms} becomes small.

ii) Increasing k_{c} causes a decrease in both N and τ_0 in a roughly inverse fashion, because we choose $k_{\text{c}} \ll k_{\text{a}} \sim k_{\text{d}}$ (see Eqs. 8 and 17). Increasing k_{c} also causes a decrease in v_{b}, especially at high k_{c}.

This is because the ratio $N_a/N_f = (k_a + k_c)/k_d$ (see Eqs. 6 and 7) increases with k_c, which leads to an increased pulling force and thus a decreased v_b. With all three factors being decreasing functions of k_c, κ_{rms} increases with k_c.

iii) Increasing k_d^0 causes a decrease in both N and τ_0, especially at low k_d^0 (see Eqs. 3, 8 and 17). v_b increases with k_d^0 because of fewer attached filaments at high k_d^0 (see Eq. 6). The overall effect is that κ_{rms} is an increasing function of k_d^0.

iv) Increasing k_n gives a linear increase in N (see Eq. 8), but has little effect on v_b (no effects on N_a/N_f, see Eqs. 6 and 7) and does not affect τ_0 (see Eq. 17), leading to a slow decreasing in κ_{rms}.

v) Increasing k_s causes a slow increase in k_d and a rapid increase in f_a (see Eqs. 3 and 10). Since N_a is inversely proportional to k_d and N_f is independent of k_d (see Eqs. 6 and 7), N_a decreases slowly as k_s increases. As a result, increasing k_s leads to a stronger resisting force from attached filaments, which decreases v_b. Because k_d is insensitive to k_s, τ_0 is also insensitive to k_s (see Eq. 17). The effect of k_s on κ_{rms} is thus very small.

vi) Increasing R leads to a linear increase in N (see Eq. 8) but has little impact on v_b and τ_0. The small decrease in v_b is probably caused by the increased chance of filaments being created at the front half of the bead, which tends to slow down the motion of the bead. The overall effect of increasing R is to cause a slow down-turn in κ_{rms}.

vii) Increasing v_0 linearly increases v_b, as expected. Increasing v_0 does not affect τ_0 but causes a decrease in N, which is due to the increased k_d (see Eqs. 3 and 6). Overall, κ_{rms} is a decreasing function of v_0 in the low velocity range, but becomes insensitive to v_0 in the high velocity range.

The above results can be subject to future experimental tests. Possible experimental approaches are: varying the surface density of N-WASP on the bead to change k_a and/or k_d^0 [12]; varying the concentration of capping proteins to change k_c; altering the concentration of Arp2/3, which is critical for the formation of branched filaments, to vary k_n; changing the concentration of cross-linking proteins to alter k_s; changing the size of bead similar to [10]; and changing the concentration of free G-actin to alter v_0.

Autocorrelation of trajectory curvature

We have checked the autocorrelation of the curvature as a function of bead's traveled distance, and find that the autocorrelation function always decays rapidly at a sub-micron distance (see Figure S12: the decay length is smaller than the window size of about 2.5 μm that we choose to calculate the local curvature). This result is quite different from the observed long-range correlation of about 10 μm [11]. One possible explanation is that in these experiments certain long-ranged spatial-temporal biases existed in the actin network at the actin-bead interface, which are not included in our model.

From the analysis in the main text, the correlation distance has to be close to $v_b\tau_0$. Then, it could be possible to get a long correlation distance with a very low value of k_c. We have tried a low value of $k_c = 0.01$ s^{-1} with which the correlation distance is expected to be about 9 μm, but our simulation still shows a correlation distance less than 2 μm. A possible explanation is that the effects of free filaments' growing away from the bead surface and being left behind the bead reduce the actual actin turn-over time τ_0, causing a much smaller correlation distance. It is also possible that certain long-range interactions exist and have not been incorporated in our mesoscopic model.

Force-Velocity Relation of Actin Networks

In this section, we consider a semi-analytical 1D model that allows to derive approximate force-velocity curves for the growing actin networks and gives insight helping to understand the simulation results reported in the main text. We also investigate how the results depend on various changes that could be made to the model. To recapitulate the results reported in the main text, the network undergoing significant disassembly in the aged gel sections recoils under a high load, reducing both net protrusion rate

of the actin network against the cantilever and the maximum force that the network can sustain. These factors cause the rapid downturn in the force-velocity relation. Another way to explain this effect is: the old, lowest part of actin pedestal starts to compress effectively creating a downward actin network flow. This flow cancels a large part of the upward polymerization speed at the network-cantilever interface, resulting in a decrease in the net rate which is equal to the polymerization rate minus recoil rate. So, the net speed of the cantilever will always be lower than the polymerization speed, unless the cantilever stops moving. Note, that the growth of the network can catch up only up to the limit because the polymerization rate is limited.

For the actin network growing against a flat cantilever, the average linear density of filaments along the obstacle is $\rho_1 = k_\mathrm{n}/k_\mathrm{c} = 20\ \mu\mathrm{m}^{-1}$ in our simulation. Due to the limitation of computation power, we choose the length of our flat obstacle to be $l = 5\ \mu$m such that the average filament number is about $\rho_1 l = 100$. To match the characteristic area density of filaments of $\rho_2 \approx 10^3\ \mu\mathrm{m}^{-2}$, the 2D network that we simulate represents a slice of 3D network with a depth of $d = \rho_1/\rho_2 \approx 0.02\ \mu$m. Therefore, the flat obstacle in our simulation corresponds to a part of cantilever tip of area $A = ld \approx 0.1\ \mu\mathrm{m}^2$. Since the actual area of the cantilever tip used in [13] is about 400 $\mu\mathrm{m}^2$, the scaling of our simulation is $0.1/400 = 1/4000$. We thus scale the spring constant of the cantilever in our simulation to be 1/4000 of the measured value of 30 pN/nm in the experiment: $k_\mathrm{lever} = 7.5$ pN/μm.

In the numerical simulations in the flat cantilever case, each of our simulations represents 180 s, which is longer than the average lifetime of a node $1/k_\mathrm{dis} \approx 120$ s. Therefore, the network disassembly is captured during the simulations. In each simulation, the value of V is sampled with 1 s interval, and then averaged with a Gaussian smooth function of 3 s width to reduce noise. F_stall is averaged over the F values at which V first becomes negative. Each data point in the force-velocity relation is averaged over at least 200 individual simulations, which limits the error to be less than 10% for each point.

For the cantilever experiment, we find $F_\mathrm{stall} \approx 20$ pN if all the filaments are pushing ($k_\mathrm{a} = 0$). Since there are about 100 filaments upon stalling, each pushing filament experiences a force of only 20/100=0.2 pN, much lower than the piconewton force that a filament can exert [3]. Therefore, the network recoils at a speed similar to that of filament elongation: $v/v_0 = \exp(-f/f_0) \approx 0.88$. Our simulated stall force scales to a stall force of only 80 nN for the actual size of cantilever used in [13], which is about 1/4 of the measured stall force of 300 nN. This discrepancy is probably resulted from a relatively high value of k_dis that we used in the simulations, which causes a high rate of network recoil and thus reduces the maximum force that the network can produce. We are unable to simulate the network for such a low k_dis because of the computation time is forbidding.

In the numerical simulations in the force-clumped bead case, each simulation represents 60 s of real time. V is measured between 30 s and 60 s with a 1 s interval, so the average age of the network during measurement is 45 s. Since 60 s is shorter than the typical disassembly time of 120 s, the network recoil does not happen. For each point in the force-velocity plot, there are at least 200 individual simulations, representing a total measurement time of 100 min for each point. We are unable to obtain V at a strong pulling force because of the small number of attached filaments used in our simulations, which leads to the rapid detachment of the bead from the network. For the bead of radius $R = 1\ \mu$m as in [14], the number of simulated filaments is about $\pi R \rho_1 \approx 60$. We use the same depth of the network $d = 0.1\ \mu$m. Thus, the network-contacting area of the bead surface in 3D is $\pi R d \approx 0.06\ \mu\mathrm{m}^2$ and the cross-section area of the network is $A \approx 2Rd \approx 0.04\ \mu\mathrm{m}^2$. Since the actual surface area of half of the bead is $2\pi R^2 \approx 6$ $\mu\mathrm{m}^2$, the scaling factor is about $0.06/6 = 1/100$.

Network length and growth velocity

The scheme of the simplified 1D model is shown in Figure S14A. Suppose that at time t, the rest length of the actin pedestal is L and its total deformation is ΔL, then the length of the compressed pedestal is

$$h = L - \Delta L. \tag{22}$$

Because the rest length of the pedestal grows at the same speed as the filaments' polymerization speed v, we have $L = \int_0^t v(t')\mathrm{d}t'$. Therefore, the cantilever's speed is

$$V = \frac{\mathrm{d}h}{\mathrm{d}t} = v - \frac{\mathrm{d}(\Delta L)}{\mathrm{d}t}. \tag{23}$$

In order to calculate ΔL, we divide the undeformed pedestal equally into M segments along its length, with $\delta = L/M$ being the length of each segment. For $M \gg 1$, the filament density ρ in each pedestal segment can be regarded as uniform. We assume that actin density ρ in each segment decreases with time as $\mathrm{d}\rho/\mathrm{d}t = -k_{\mathrm{dis}}\rho$, where k_{dis} is the disassembly rate of the network. Then, the density of filaments in segment-i ($i = 1, ..., M$) should decrease exponentially with the segment's lifetime τ_i: $\rho_i \propto e^{-k_{\mathrm{dis}}\tau_i}$. The network's Young's modulus is estimated to scale as $\rho^{2.2} - \rho^{2.5}$, depending on the property of the network [15, 16], so we choose the power of 2.5 assuming the network is densely cross-linked. Then, the Young's modulus for segment i can be written as $Y_i = Ye^{-k\tau_i}$, where Y is the Young's modulus of a newly created network and $k = 2.5k_{\mathrm{dis}}$ is a constant. Thus the spring constant of segment i is $k_{\mathrm{s},i} = Y_i A/\delta = (YA/\delta)e^{-k\tau_i}$. When the network is compressed by an external force F, the deformation of each segment Δl_i should be inversely proportional to its spring constant, but since segments cannot have negative lengths, Δl_i should also be limited by δ: $\Delta l_i = \min(F/k_{\mathrm{s},i}, \delta) = \min(\alpha\delta e^{k\tau_i}, \delta)$, where $\alpha = F/YA$. Therefore the total deformation of the network is

$$\Delta L = \sum_{i=1}^{M} \Delta l_i = m\delta + \alpha\delta \sum_{i=m+1}^{M} e^{k\tau_i} = x^* + \alpha\delta \sum_{i=m+1}^{M} e^{k\tau_i}, \tag{24}$$

where m is the number of segments with deformations being the same as δ, and $x^* = m\delta$ is the rest length of these segments. It is easy to find that if m is nonzero, it satisfies the equation $\alpha e^{k\tau_m} = 1$, which leads to the definition of the characteristic lifetime of the network segments

$$\tau^* = \tau_m = -(\ln\alpha)/k. \tag{25}$$

Network segments with lifetimes greater or equal to τ^* will collapse to zero length due to compression.

In the continuum limit, we define the lifetime of a network segment at time t to be

$$\tau(x, t) = t - t_{\mathrm{c}}(x), \tag{26}$$

where x is the location of the segment in the undeformed network and $t_{\mathrm{c}}(x) = \int_0^x \mathrm{d}x'/v(x')$ is the creation time of the segment. Eq. 24 then becomes

$$\Delta L = x^* + \alpha \int_{x^*}^{L} e^{k\tau(x,t)}\mathrm{d}x = x^* + \alpha e^{kt} \int_{x^*}^{L} e^{-kt_{\mathrm{c}}(x)}\mathrm{d}x. \tag{27}$$

The value of x^* depends on the history of external force.

i) Constant F. We assume that the number of working filaments, N, is a constant. So, both the force per working filament, F/N, and the velocity of polymerization, v, are constants. Thus we have $t_{\mathrm{c}}(x) = x/v$ and $L = vt$. For $t < \tau^*$, x^* should be 0, because no segments collapse to zero length. For $t \geq \tau^*$, x^* can be obtained from Eq. 26: $\tau^* = t - t_{\mathrm{c}}(x^*) = t - x^*/v$. Therefore, x^* can be written as

$$x^* = \begin{cases} 0 & \text{if } t < \tau^*, \\ v(t - \tau^*) & \text{if } t \geq \tau^*. \end{cases} \tag{28}$$

Eq. 27 then becomes

$$\Delta L = \begin{cases} (\alpha v/k)\big(e^{kt} - 1\big) & \text{if } t < \tau^*, \\ L - h^* & \text{if } t \geq \tau^*, \end{cases} \tag{29}$$

where

$$h^* = (v/k)(\alpha - \ln\alpha - 1) \tag{30}$$

is time-independent. From Eqs. 22, 23 and 29 we obtain

$$h(t) = \begin{cases} vt - (\alpha v/k)\big(e^{kt} - 1\big) & \text{if } t < \tau^*, \\ h^* & \text{if } t \geq \tau^*, \end{cases} \tag{31}$$

$$V(t) = \begin{cases} \big(1 - \alpha e^{kt}\big)v & \text{if } t < \tau^*, \\ 0 & \text{if } t \geq \tau^*. \end{cases} \tag{32}$$

Eq. 31 shows that h increases linearly with t in the initial stage, and then reaches a steady-state value h^* at τ^* when the network starts collapsing. The plots of F-dependence of initial V and the h-t relation are shown in Figure 5D in the main text and Figure S14C, respectively.

ii) Variable F. If F is proportional to the deflection of the cantilever, then

$$F = k_{\text{lever}}\Delta h, \tag{33}$$

where $\Delta h = h - h_{\text{min}}$ is the deflection of the cantilever with h_{min} being the initial length of the actin pedestal. Then v varies with time and $t_{\text{c}}(x)$ depends on the loading history of the cantilever. For $t < \tau^*$, because $x^* = 0$, we write Eq. 27 as

$$\Delta L = \alpha B, \tag{34}$$

where $B = \int_0^L e^{k\tau(x,t)}\mathrm{d}x$. From Eq. 26 and the fact that $\partial L/\partial t = v$ and $t_{\text{c}}(L) = t$, B satisfies the equation:

$$\frac{\mathrm{d}B}{\mathrm{d}t} - kB = v. \tag{35}$$

From Eqs. 23, 33-35, we obtain the following relation for V:

$$\frac{\mathrm{d}V}{\mathrm{d}t} = \frac{\mathrm{d}^2 h}{\mathrm{d}t^2} = \frac{(k\Delta h + 2V)[k_{\text{lever}}vV + kYA(v - V)] + (k\Delta h + V)(k_{\text{lever}}\Delta h - YA)(\partial v/\partial t)}{k_{\text{lever}}v\Delta h - YA(k\Delta h + v)}. \tag{36}$$

The term $\partial v/\partial t$ in the above equation can be evaluated through

$$\frac{\partial v}{\partial t} = \frac{\partial v}{\partial F}\frac{\partial F}{\partial h}\frac{\partial h}{\partial t} = \frac{k_{\text{lever}}V}{N}\frac{\partial v}{\partial f} = -\frac{k_{\text{lever}}vV}{Nf_0}, \tag{37}$$

where $f = F/N$ is the average force per filament and $v = v_0\exp(-f/f_0)$ is the force-velocity relation of individual filaments. From Eqs. 23, 33, 36 and 37, we have

$$\begin{aligned} \frac{\mathrm{d}V}{\mathrm{d}F} &= \frac{\partial V}{\partial t}\frac{\partial t}{\partial F} = \frac{1}{k_{\text{lever}}V}\frac{\mathrm{d}V}{\mathrm{d}t} \\ &= \frac{(\beta F + 2V)[k_{\text{lever}}vV + kYA(v - V)] + (\beta F + V)(YA - F)(k_{\text{lever}}vV/Nf_0)}{k_{\text{lever}}V[Fv - YA(\beta F + v)]}, \end{aligned} \tag{38}$$

where $\beta = k/k_{\text{lever}}$ is a constant. Then, function $h(t)$ and the force-velocity relation can be obtained numerically from Eqs. 36 to 38 (see Figure S14D). For the case of $YA \gg F \approx Nf_0$ and $\beta F \gg v \approx v_0 \gg V$, Eq. 38 can be approximated as $\mathrm{d}V/\mathrm{d}F \approx -\beta v_0/V$, which gives the approximate force-velocity relation

$$V \approx V_0\sqrt{1 - F/F_{\text{stall}}}, \tag{39}$$

where V_0 is the network's velocity at $F = 0$ and $F_{\text{stall}} = V_0^2/2\beta v_0$ is the stall force (see Figure 5C in the main text). Parameter V_0 can be calculated as follows: right before the pedestal starts pushing against the

cantilever, it has length $L = h_{\min}$ in the undeformed state. Assuming that the network grows at a constant speed v_0 before deflecting the cantilever, then $B = B_0 = \int_0^L \exp(kx/v_0)\mathrm{d}x = [\exp(kh_{\min}/v_0) - 1]v_0/k$. From Eqs. 23 and 34, one finds $V_0 = v_0/(1 + k_{\mathrm{lever}}B_0/YA)$.

Eqs. 34-38 are valid only for $t < \tau^*$. One can find that the network will reach a steady state when t is sufficiently large. Defining $F^* = k_{\mathrm{lever}}(h^* - h_{\min})$ and $\alpha^* = F^*/YA$ with h^* being the steady state position of the cantilever, Eq. 30 becomes

$$h^* = (v^*/k)(\alpha^* - \ln \alpha^* - 1), \tag{40}$$

where $v^* = v(F^*/N)$ is determined by the force-velocity relation of single filaments. Then, h^* can be found numerically from the above equation with a root-finding algorithm. Eq. 25 gives $\tau^* = -(\ln \alpha^*)/k$, which is roughly the time required for the network to reach the steady state. A simple way to explain the predicted effect: the old, lowest part of actin pedestal starts to compress effectively creating downward actin network flow. This flow cancels a large part of the upward polymerization at the network-cantilever interface. So, the net rate with which the network protrudes and pushes the cantilever decreases. Therefore, at a given force, which determines the polymerization rate, the net protrusion speed will be lower than the polymerization speed.

Kymograph

Based on the above calculations, we predict the time-lapse plot of the positions of actin network's material points, or, the kymograph of the actin speckles (Figure S14B). We find a time scale t^* and a length scale h^* such that the network grows continuously for $t < t^*$ until reaching h^* at $t \approx t^*$ (Figure S14, C and D). For $t > t^*$, the overall length of the actin pedestal will reach a steady state. For the force-clamping experiment, we find $t^* = -\ln(\alpha)/k$ and $h^* = (v/k)(\alpha - \ln \alpha - 1)$, where $k = 2.5k_{\mathrm{dis}}$ is the decay rate constant of the Young's modulus of the network, $\alpha = F/YA$ and v is the average filament growth speed. For the cantilever experiment, t^* and h^* have similar formulas, but since α is no longer a constant, t^* and h^* can be found numerically with a root-finding algorithm.

i) Constant F. We assume that at time t_0, a network component P is created underneath the cantilever with $h = h_{\mathrm{P}}^0$ (see Figure S14B). Because the rest length of the pedestal at t_0 is $L_{\mathrm{P}} = vt_0$, h_{P}^0 can be obtained from Eq. 31 as

$$h_{\mathrm{P}}^0 = h(t_0) = \begin{cases} vt_0 - (\alpha v/k)\left(e^{kt_0} - 1\right) & \text{if } t_0 < \tau^*, \\ h^* & \text{if } t_0 \geq \tau^*. \end{cases} \tag{41}$$

Therefore, for $t_0 < \tau^*$, we have

$$t_0 = h_{\mathrm{P}}^0/v - (\alpha + w_{\mathrm{P}})/k, \tag{42}$$

where $w_{\mathrm{P}} = \mathrm{W}[-\alpha \exp(kh_{\mathrm{P}}^0/v - \alpha)]$ with $\mathrm{W}(x)$ being the Lambert W function satisfying the equation $x = \mathrm{W}(x)e^{\mathrm{W}(x)}$.

For $t_0 \leq \tau^*$, at $t = t_0 + \tau_{\mathrm{P}}$ when the lifetime of the network at P is τ_{P}, the deformation of the network between the bottom and P can be obtained from Eq. 27 as

$$\begin{aligned} \Delta L_{\mathrm{P}}(t) &= x^* + \alpha e^{kt} \int_{x^*}^{L_{\mathrm{P}}} e^{-kx/v}\mathrm{d}x, \\ &= \begin{cases} (\alpha v/k)\left(e^{kt} - e^{k\tau_{\mathrm{P}}}\right) & \text{if } t < \tau^*, \\ v(t - \tau^*) + (\alpha v/k)\left(e^{k\tau^*} - e^{k\tau_{\mathrm{P}}}\right) & \text{if } \tau^* \leq t < t_0 + \tau^*, \\ L_{\mathrm{P}}(t) & \text{if } t \geq t_0 + \tau^*. \end{cases} \end{aligned} \tag{43}$$

From Eqs. 25, 41-43, the position of P as a function of τ_P can be found as

$$\begin{aligned} h_\mathrm{P}(\tau_\mathrm{P}) &= L_\mathrm{P} - \Delta L_\mathrm{P} \\ &= \begin{cases} h_\mathrm{P}^0 - (v/k)(\alpha + w_\mathrm{P})\big(1 - e^{k\tau_\mathrm{P}}\big) & \text{if } \tau_\mathrm{P} < \tau^* - t_0, \\ v(\tau^* - \tau_\mathrm{P}) - (\alpha v/k)\big(e^{k\tau^*} - e^{k\tau_\mathrm{P}}\big) & \text{if } \tau^* - t_0 \le \tau_\mathrm{P} < \tau^*, \\ 0 & \text{if } \tau_\mathrm{P} \ge \tau^*. \end{cases} \end{aligned} \tag{44}$$

Noting that for $\tau_\mathrm{P} \ge \tau^*$, the network below point P collapses to 0. From Eq. 44, the velocity of point P can be obtained as

$$V_\mathrm{P}(\tau_\mathrm{P}) = \frac{\mathrm{d}h_\mathrm{P}}{\mathrm{d}\tau_\mathrm{P}} = \begin{cases} (\alpha + w_\mathrm{P})e^{k\tau_\mathrm{P}} v & \text{if } \tau_\mathrm{P} < \tau^* - t_0, \\ \big(\alpha e^{k\tau_\mathrm{P}} - 1\big)v & \text{if } \tau^* - t_0 \le \tau_\mathrm{P} < \tau^*, \\ 0 & \text{if } \tau_\mathrm{P} \ge \tau^*. \end{cases} \tag{45}$$

For $t_0 > \tau^*$, the network is at steady state. Therefore $h_\mathrm{P}(\tau_\mathrm{P})$ and $V_\mathrm{P}(\tau_\mathrm{P})$ should be the same as those for $t_0 = \tau^*$:

$$h_\mathrm{P}(\tau_\mathrm{P}) = \begin{cases} v(\tau^* - \tau_\mathrm{P}) - (\alpha v/k)\big(e^{k\tau^*} - e^{k\tau_\mathrm{P}}\big) & \text{if } \tau_\mathrm{P} < \tau^*, \\ 0 & \text{if } \tau_\mathrm{P} \ge \tau^*, \end{cases} \tag{46}$$

$$V_\mathrm{P}(\tau_\mathrm{P}) = \begin{cases} \big(\alpha e^{k\tau_\mathrm{P}} - 1\big)v & \text{if } \tau_\mathrm{P} < \tau^*, \\ 0 & \text{if } \tau_\mathrm{P} \ge \tau^*. \end{cases} \tag{47}$$

The kymograph of a 1D network with $F = 100$ nN is shown in Figure S14C.

ii) Variable F. For F being proportional to the cantilever deflection, it is difficult to derive an analytical form for the motion of a material point in the actin network. We use 1D node-spring simulations to obtain the kymograph shown in Figure S14D. Similar to the case of constant F, h increases with t for $t < \tau^*$ and reaches a steady state for large t, but it has an overshoot near $t = \tau^*$ before reaching the steady state. This is caused by the fast actin growth at low forces, which produces a longer pedestal than what is expected at a constant force. One can also see that for $t < \tau^*$, speckle trajectories at variable force descend more rapidly than those at constant force because, as the force increases, it compresses the actin network more than a constant force does. At the steady state, however, the speckle trajectories have the same shape as those at constant force.

Effects on individual filament force-velocity relations, attachments, geometry and stiffness

We have examined effects of the exact form of the filament force-velocity relation, attachment rate, surface geometry and actin network stiffness on the resulting force-velocity relation of the actin network. For simplicity, we consider only the experiment with the force-clamping setup.

We start with a simple case in which all filaments are pushing, not attached (by setting $k_\mathrm{a} = 0$) and growing against a flat obstacle from the boundary of the infinitely stiff network. To see how the shape of the force-velocity relation of individual pushing filaments affects the shape of the force-velocity relation of the network, we have tried both concave-up and concave-down shapes for the force-velocity relation (see Figure S1). These two shapes represent the predictions from the Brownian ratchet theory [3] and the end-tracking theory [17,18], respectively. The simulated network's force-velocity relation under these two conditions is shown in Figure S15A. We find that the force-velocity relation of the network has a similar shape to that of the individual filaments, indicating that forces are shared roughly equally among pushing filaments. This is confirmed by the simulation results showing that the force always has a very narrow distribution (half-width < 0.5 pN) before the network is stalled (Figure S15, B and C).

Next, we investigate the effect of filament attachments by setting $k_a = 1\ s^{-1}$. We find that the shape of the network's force-velocity relation becomes insensitive to the shape of the force-velocity relation of individual pushing filaments (see Figure S15D), similar to the previous findings [9]. There are two factors contributing to the shape of the force-velocity relation. One is that attached filaments pull more strongly in the high velocity regime but less so in the low velocity regime, causing a more significant reduction in velocity at low forces. The other factor is that the forces exerted by the pushing filaments have a much broader distribution (see Figure S15, E and F), smothering the effect of shape of the force-velocity relation of individual filaments. We hypothesize that the broadening of the force distribution of is caused by the dynamic conversion between the states free and attached filaments. As free filaments elongate toward the obstacle, they are compressed by the obstacle if the filaments make contact. When free filaments later become attached to the obstacle, they stop polymerizing but keep their compressions. As the net growth of the filament network continues to displace the obstacle, the initial compression in the attached filaments is gradually relieved until the filaments detach again. This dynamic conversion between the deformation states of free and attached states thus creates a broad distribution of forces. We have also noticed that networks with a concave-down-shaped force-velocity relation for single filaments tend to have a wider distribution of forces than those with a concave-up-shaped force-velocity relation. The reason is that for filaments with a concave-down-shaped force-velocity relation, their growth velocity is insensitive to changes in force when the force is less than the stall force. Therefore, at a given velocity, pushing filaments with a concave-down-shaped force-velocity relation have a wider range of values of forces than those with a concave-up-shaped force-velocity relation.

To see the effect of the surface geometry, we replace the flat surface with a spherical one in two simulations described above and find that the shapes of the force-velocity relations under the considered conditions are similar to those corresponding to the flat surface. Therefore, the geometry of the surface does not play an important role in the shape of the force-velocity relation of the network. We also notice that the velocity tends to be high near $F = 0$ (see Figure S15, G and J). The reason is that at $F = 0$ the pushing filaments on the two sides of the spherical surface generate a strong squeezing effect, which causes a much higher velocity. This squeezing effect is rapidly suppressed when the force increases. The squeezing effect can also be observed from the biased distribution of forces toward the high values (see Figure S15, H–I and K–L).

For a deformable network with a fixed disassembly rate and both pushing and attached filaments, the compression of the actin pedestal underneath the obstacle slows down the net protrusion of the network, especially at high forces. Therefore, velocity decreases more rapidly with force at high force, causing a slightly concave-up force-velocity relation for both flat and spherical surfaces (see Figure S15, M and P). The stall force for the deformable actin network is also lower than that for the stiff network. The shape of the force-velocity relation of individual pushing filaments, however, does not have strong impact on the shape of the force-velocity relation of the network, similar to the results for the stiff networks. From the distribution of forces for individual pushing filaments (see Figure S15, N–O and Q–R), we find that the force on the pushing filaments in deformable networks is generally lower than that in stiff networks, even near the stall. This shows that the stall of a deformable network's growth does not necessarily represent the stall of all pushing filaments; it could also indicate a dynamic balance between the network growth and network compression and/or disassembly. Figure S16 shows the simulations' snapshots in which the structures of the model actin network, as well as stress and strain distributions, can be seen.

References

1. Zimmermann J, Enculescu M, Falcke M (2010) Leading-edge-gel coupling in lamellipodium motion. Phys Rev E 82: 051925.

2. Peskin CS, Odell GM, Oster GF (1993) Cellular motions and thermal fluctuations: the Brownian ratchet. Biophys J 65: 316-324.

3. Mogilner A, Oster G (2003) Force generation by actin polymerization II: the elastic ratchet and tethered filaments. Biophys J 84: 1591-1605.

4. Wyart M, Liang H, Kabla A, Mahadevan L (2008) Elasticity of floppy and stiff random networks. Phys Rev Lett 101: 215501.

5. Boal D (2002) Mechanics of the Cell. Cambridge, UK: Cambridge University Press.

6. Plastino J, Olivier S, Sykes C (2004) Actin filaments align into hollow comets for rapid VASP-mediated propulsion. Curr Biol 14: 1766-1771.

7. Risca VI, Wang EB, Chaudhuri O, Chia JJ, Geissler PL, et al. (2012) Actin filament curvature biases branching direction. Proc Natl Acad Sci USA 109: 2913-2918.

8. Lacayo CI, Soneral PAG, Zhu J, Tsushida MA, Footer MJ, et al. (2012) Choosing orientation: influence of cargo geometry and ActA polarization on actin comet tails. Mol Biol Cell 23: 614-629.

9. Zhu J, Carlsson AE (2010) Effects of molecular-scale processes on observable growth properties of actin networks. Phys Rev E 81: 031914.

10. Schmidt S, van der Gucht J, Biesheuvel PM, Weinkamer R, Helfer E, et al. (2008) Non-Gaussian curvature distribution of actin-propelled biomimetic colloid trajectories. Eur Biophys J 37: 1361-1366.

11. Shaevitz JW, Fletcher DA (2008) Curvature and torsion in growing actin networks. Phys Biol 5: 026006.

12. Co C, Wong DT, Gierke S, Chang V, Taunton J (2007) Mechanism of actin network attachment to moving membranes: barbed end capture by N-WASP WH2 domains. Cell 128: 901-913.

13. Parekh SH, Chaudhuri O, Theriot JA, Fletcher DA (2005) Loading history determines the velocity of actin-network growth. Nat Cell Biol 7: 1219-1223.

14. Marcy Y, Prost J, Carlier MF, Sykes C (2004) Forces generated during actin-based propulsion: a direct measurement by micromanipulation. Proc Natl Acad Sci USA 101: 5992-5997.

15. de Gennes PG (1979) Scaling Concepts in Polymer Physics. Ithaca: Cornell University Press, 1st edition.

16. MacKintosh FC, Käs J, Janmey PA (1995) Elasticity of semiflexible biopolymer networks. Phys Rev Lett 75: 4425.

17. Dickinson RB, Purich DL (2002) Clamped-filament elongation model for actin-based motors. Biophys J 82: 605-617.

18. Dickinson RB, Caro L, Purich DL (2004) Force generation by cytoskeleton filament end-tracking proteins. Biophys J 87: 2838-2854.

Table

Table S1. Model variables and parameters.

Symbol	Definition	Value	References
$k_{\rm a}$	Filament attachment rate constant	1 s^{-1}	assumed
$k_{\rm c}$	Filament capping rate constant	0.1 s^{-1}	assumed
$k_{\rm d}^0$	Filament detachment rate at zero-force	2.7 s^{-1}	assumed
$k_{\rm n}$	Filament nucleation rate	2 $\mu m^{-1} s^{-1}$	assumed
$k_{\rm s}$	Spring constant of filament attachment	300 pN/μm	assumed
$k_{\rm dis}$	Network disassembly rate	8×10^{-3} s^{-1}	assumed
k	Decay rate constant of Young's modulus	$2.5 k_{\rm dis}$	(11, 12)
a	Bead's short-axis	0.5 μm	(2)
b	Bead's long-axis	1 μm	(2)
v_0	Filament's free-polymerization speed	50 nm/s	assumed
f_0	Force scale	1.5 pN	(3)
$k_{\rm lever}$	Spring constant of cantilever	30 pN/nm (in analytic calc.)<br>7.5 pN/μm (in simulations)	(9)
A	Network's cross-section area	400 μm^2 (in analytic calc.)<br>0.1 μm^2 (in simulations)	(9)
Y	Young's modulus of nascent network	10 kPa (in analytic calc.)<br>3 kPa (in simulations)	(9)
$k_{\rm d}$	Average filament detachment rate	varies	
v	Filament's average growth velocity	varies	
$v_{\rm b}$	Bead velocity	varies	
V	Cantilever's velocity	varies	
L	Network's rest length	varies	
ΔL	Total network deformation	varies	
h	Length of deformed network	varies	
h^*	Steady-state length of deformed network	varies	
x^*	Rest length of collapsed network	varies	
$t_{\rm c}$	Creation time of a network segment	varies	
τ	Lifetime of a network segment	varies	
τ^*	Characteristic lifetime of a network segment	varies	
τ_0	Turn-over time of actin filaments	varies	

Model variables and parameters.

Figures

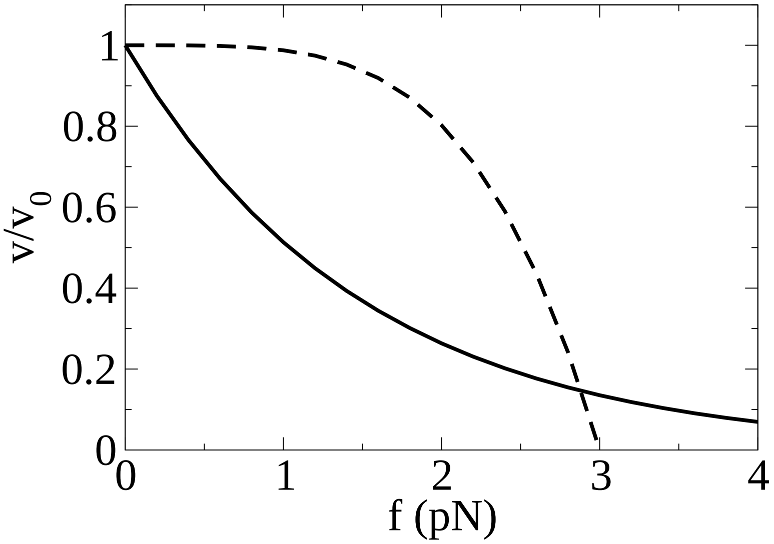

Figure S1. Hypothesized possible shapes of the force-velocity relation for individual actin filaments. Solid line: concave-up shape with $v/v_0 = \exp(-f/f_0)$. Dashed line: concave-down shape with $v/v_0 = 1 - (f/f_s)^4$, where $f_s = 3$ pN is the stall force.

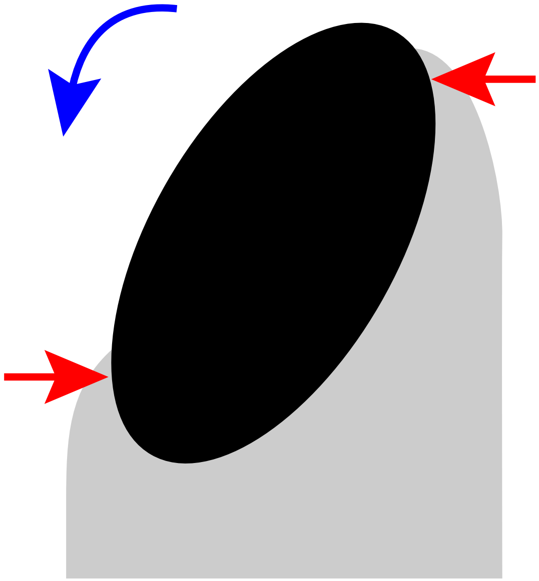

Figure S2. Schematic of the elastic model. Squeezing forces (red arrows) from an elastic actin tail (gray) tend to rotate an elliptic bead (black) to move along its long-axis (blue arrow).

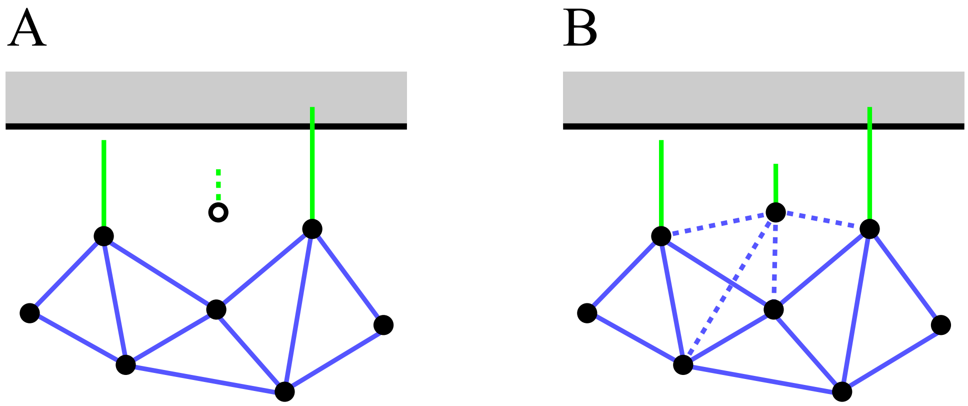

Figure S3. Schematic of filament nucleation in the mesoscopic model. Actin filaments (green lines) are anchored to the node-spring network (blue lines and black solid circles) at their pointed ends. (A) A new filament (green dashed line) is created near the bead surface (gray rectangle) with pointed end represented by a black open circle. (B) The nascent filament is anchored to the network by connecting its pointed end to 4 nearby nodes with springs (dashed blue lines), which also expand the network.

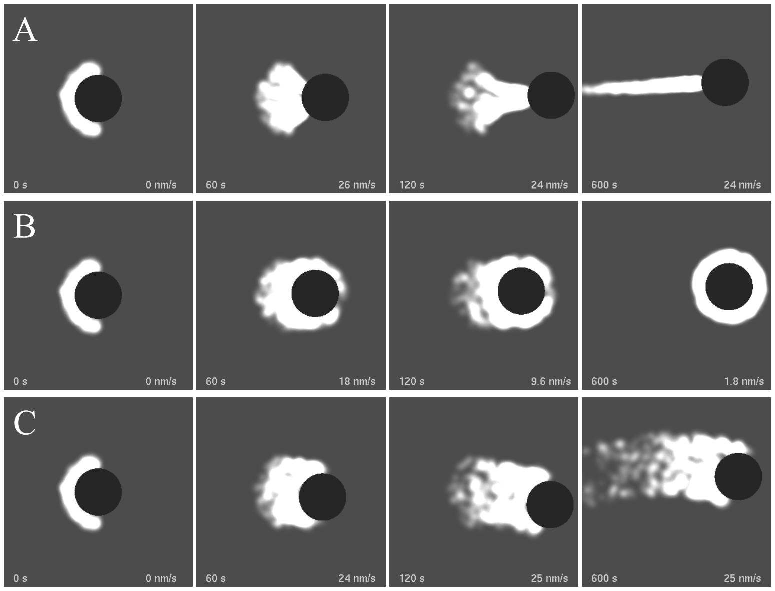

Figure S4. Simulation snapshots of actin-propelled spherical beads (black circles) with $R = 1$ μm. White indicates the density of pointed ends of actin filaments. (A) Filaments are created via branching. (B) Filaments are created via spontaneous nucleation. (C) Filaments are created via both branching and spontaneous nucleation. The total branching rate is chosen to be the same as the total rate of spontaneous nucleation.

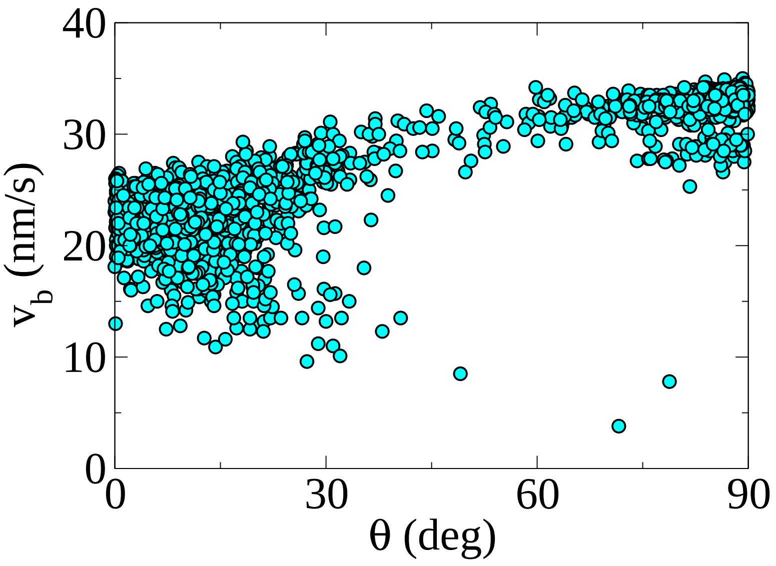

Figure S5. Scatter plot of simulated beads' speed vs. orientation angle (between the direction of propulsion and long axis). The lengths of the short and long axes of ellipsoidal beads are $a = 0.5$ μm and $b = 1$ μm, respectively. Circles: 2000 data points taken from 100 individual simulations each representing 10^4 s of real time.

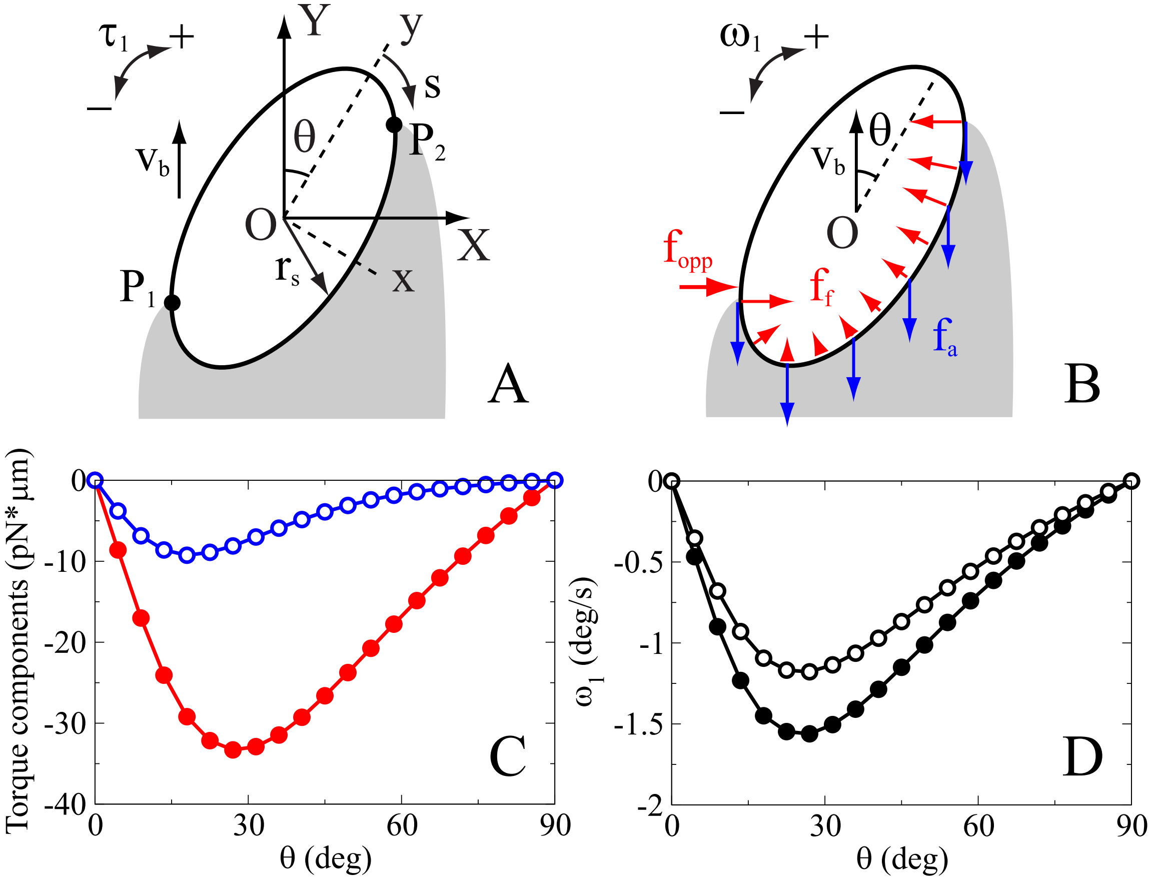

Figure S6. Torque-induced rotation of bead. (A) Schematic of the bead (ellipse) and its tail (gray). (B) Schematic of the distribution of pushing (red) and pulling (blue) forces along the bead surface. (C) Calculated torques (solid red circles: from free filaments' pushing and additional opposing force, open blue circles: from attached filaments' pulling). (D) Calculated torque-induced angular velocity with mobility coefficients of $c = 0.53$ (solid circles) and $c = 0.4$ (open circles).

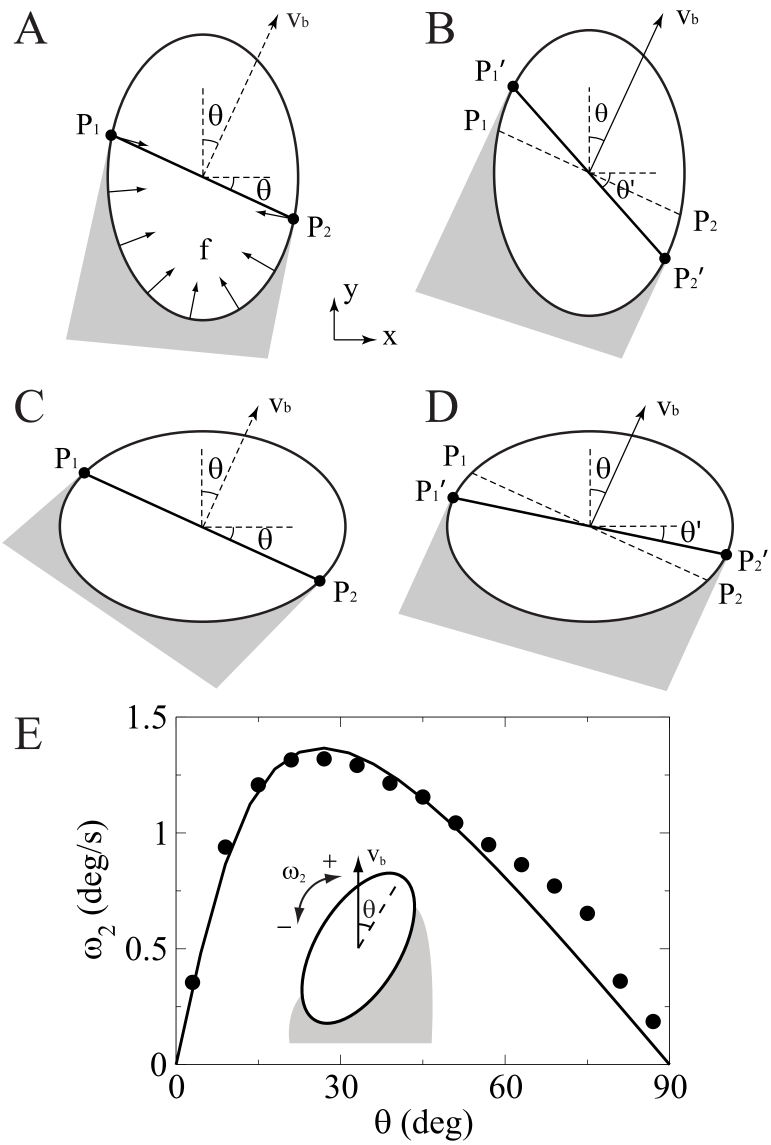

Figure S7. Tail-reorientation-induced rotation of the bead with respect to its tail. (A–D) Schematics of the elliptic bead pushed by uniformly distributed filaments in the bead's frame-of-reference. The pushing forces are perpendicular to the bead surface. (A) Initially, the bead is moving roughly along its long-axis. The bead-tail boundary P_1P_2 is tilted by θ from the bead's short-axis. The direction of the bead velocity v_b is then determined by the total pushing force from the filaments. (B) After the bead moves, the tail remodels and forms new contact region behind the bead, resulting in new bead-tail boundary $P'_1P'_2$ with $\theta' > \theta$ (unstable orientation). (C–D) The bead moves roughly along its short-axis, resulting in $\theta' < \theta$ (stable orientation). (E) Approximated (line) and simulated (circles) ω_2-θ relation. Inset: schematic of bead's rotation in the tail's frame-of-reference.

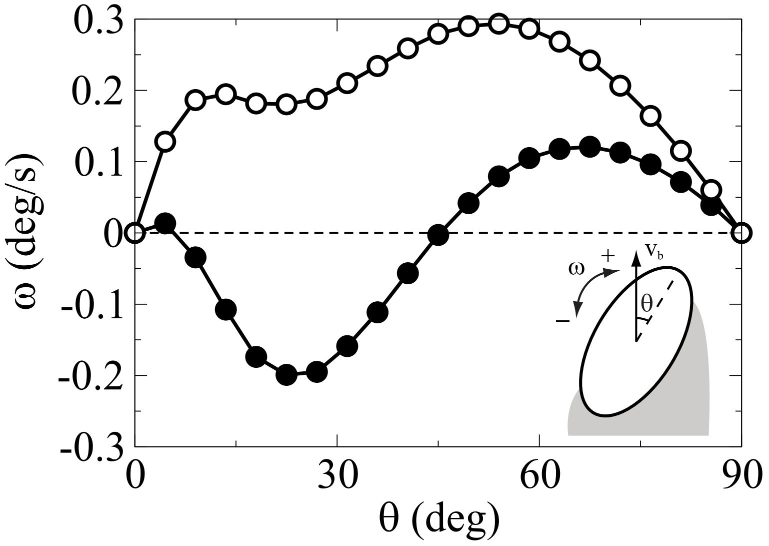

Figure S8. Calculated total angular velocity of the bead with respect to its tail as a function of θ. The mobility coefficients of the beads are 0.53 (solid circles) and 0.4 (open circles). Inset: schematic of bead's rotation in the tail's frame-of-reference.

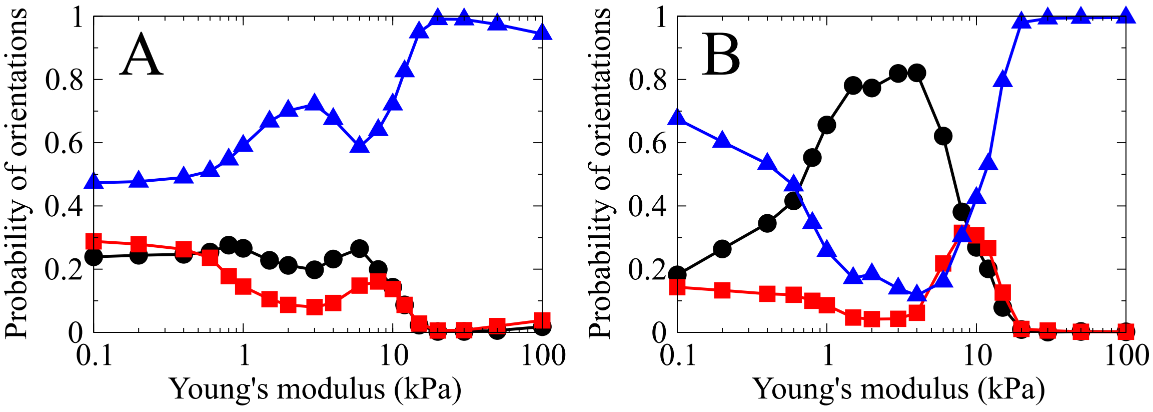

Figure S9. Simulated probability distribution of beads' orientation as a function of Young's modulus of actin networks. Bead has an aspect ratio of 1.5 (A) and 2.5 (B). Black circles: bead moves along the long-axis ($0 \leq \theta < 30°$). Red squares: bead moves at a skewed orientation ($30° \leq \theta < 60°$). Blue triangles: bead moves along the short-axis ($60° \leq \theta < 90°$).

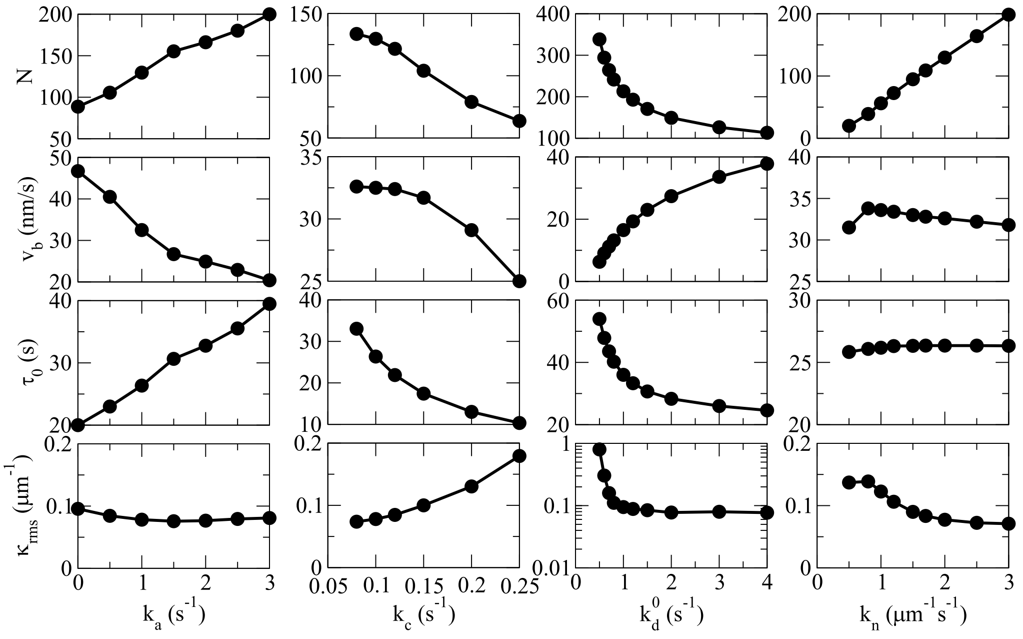

Figure S10. Simulated variables N, v_b, τ_0 and κ_{rms} as functions of parameters k_a, k_c, k_d^0 and k_n.

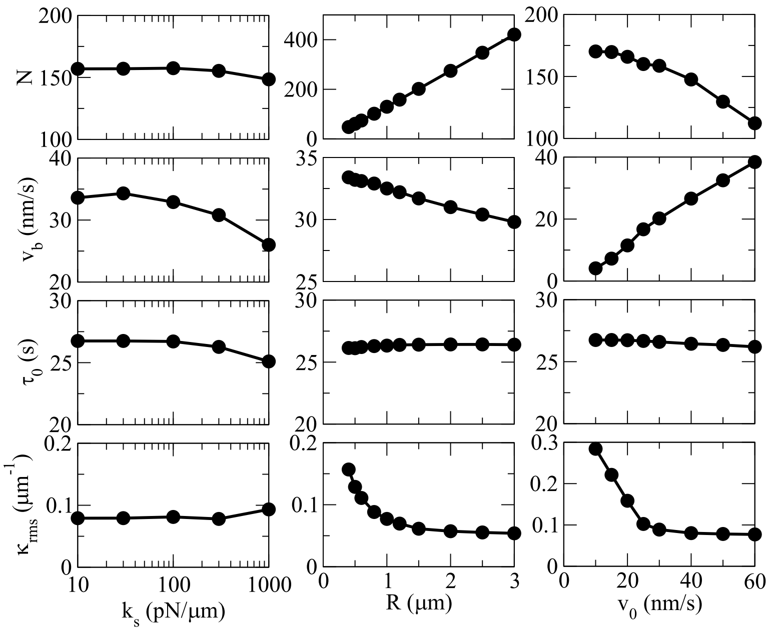

Figure S11. Simulated variables N, v_b, τ_0 and κ_{rms} as functions of parameters k_s, R and v_0.

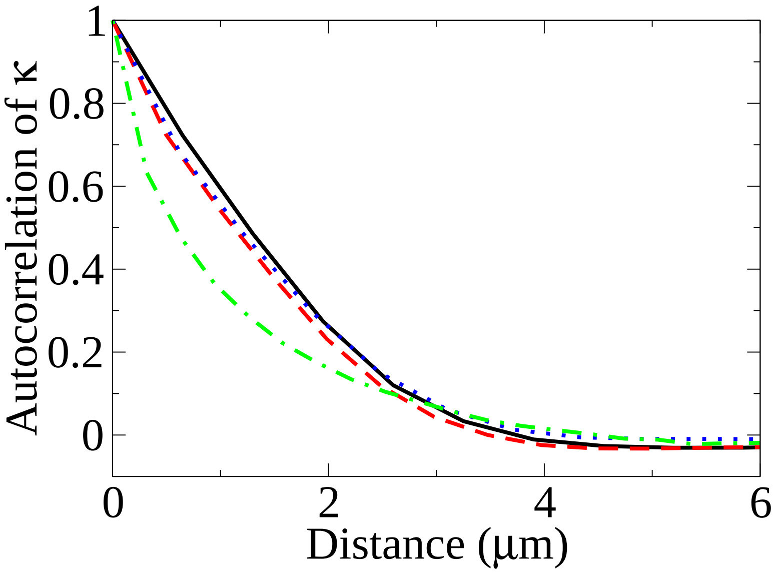

Figure S12. Simulated autocorrelation of the trajectory curvature, κ, vs. traveled distance. Solid black line: with default values of parameters. Dashed red line: doubling k_{a}. Dotted blue line: doubling R. Dot-Dashed green line: $k_{\mathrm{d}}^{0} = 0.5\ \mathrm{s}^{-1}$.

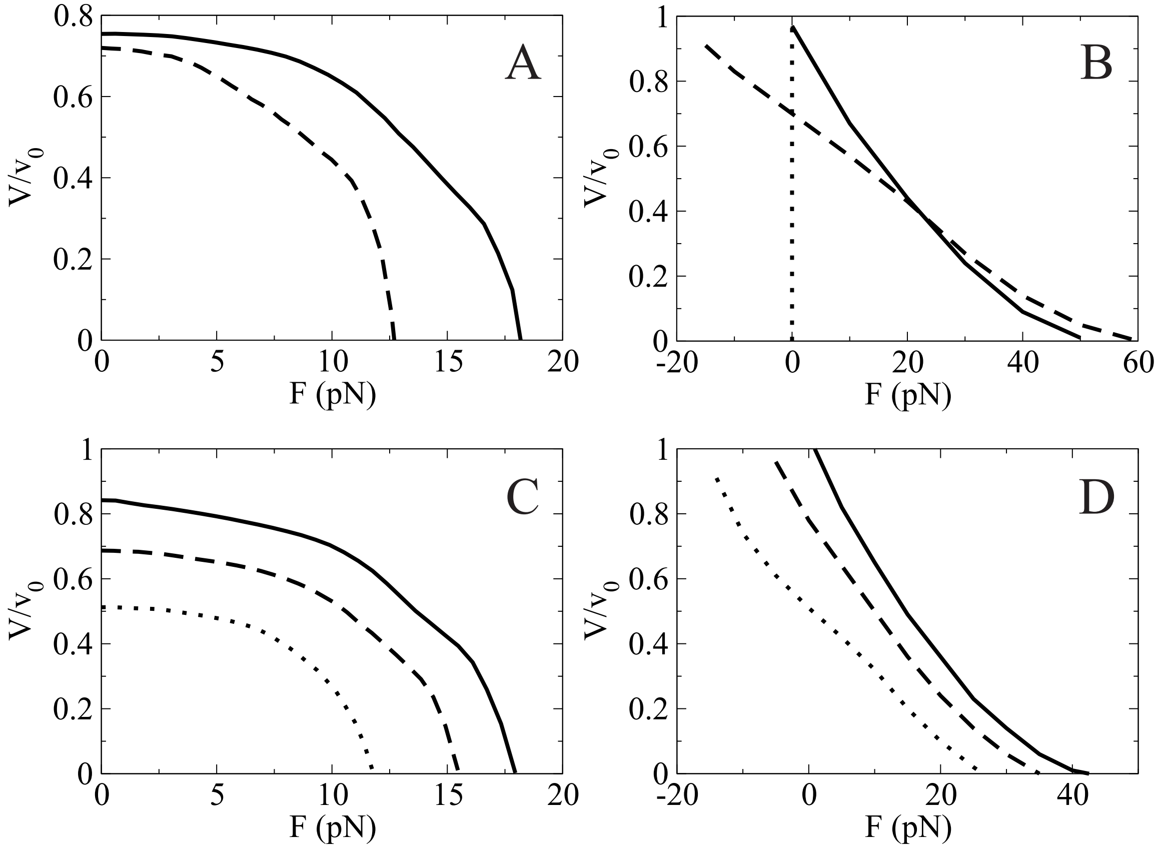

Figure S13. Simulated force-velocity relation. (A) Similar to the setup in Figure 4A of the main text, but with a concave-down shaped force-velocity relation for individual pushing filaments (solid line), or with an obstacle geometry as in Figure 4B of the main text (dashed line). (B) Similar to the setup in Figure 4B, but with a concave-down shaped force-velocity relation for individual pushing filaments (solid line), or with an obstacle geometry as in Figure 4A (dashed line). (C) Cantilever experiment with a flat surface and $N_a/N_f = 0.18$ (solid line), 0.37 (dashed line) and 0.74 (dotted line). (D) Force-clamping experiment with a spherical surface and $N_a/N_f = 0.18$ (solid line), 0.37 (dashed line) and 0.74 (dotted line).

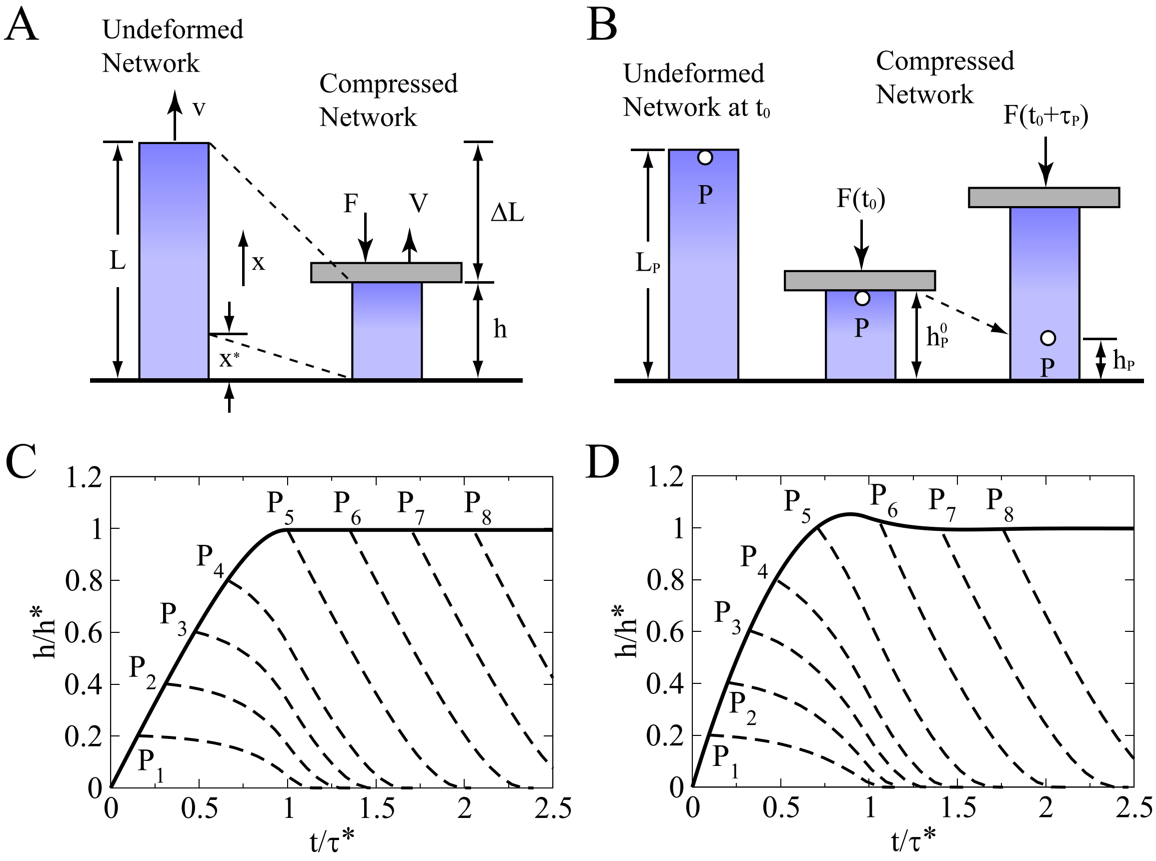

Figure S14. Calculated kymograph of actin gels under load. (A and B) Schematic of the 1D network model. (A) Actin pedestal (blue) is compressed by flat cantilever (grey) with force F. In the undeformed state, the pedestal has length L and grows with velocity v. Under compression, the network has length h and grows with velocity V. Network segment of length x^* at the bottom of the undeformed network is compressed to zero-length because of its low stiffness. (B) Motion of a speckle P (white circles) in the network. From left to right: undeformed network at $t = t_0$, compressed network at $t = t_0$, and compressed network at $t = t_0 + \tau_P$. Point P moves down from t_0 to $t_0 + \tau_P$ due to the recoil of the network below it. (C and D) Calculated coordinate of the leading edge of the actin network (solid lines) and traces of 8 speckles (dashed lines, P_1-P_8) as functions of t for a constant force (C) and for a varying force with the magnitude being proportional to the cantilever displacement (D).

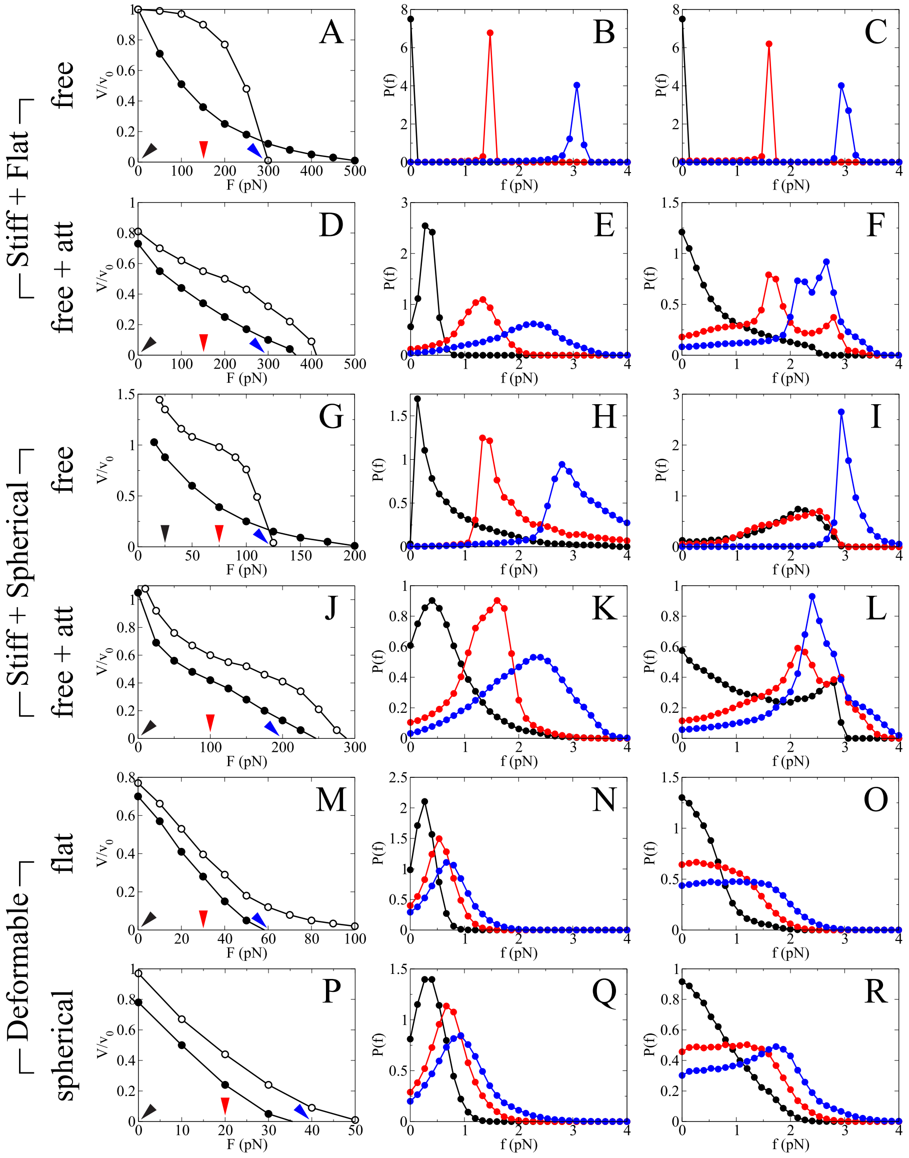

Figure S15. Force-velocity relation and distribution of forces from force-clamping simulations. Left column: force-velocity relation. Solid circles: pushing filaments have a concave-up force-velocity relation (as in Figure S1). Open circles: pushing filaments have a concave-down force-velocity relation. Middle and right columns: distribution of forces from pushing filaments that have either a concave-up force-velocity relation (middle column) or a concave-down force-velocity relation (right column). Colors indicate different values of force, which are marked with arrowheads of the same colors in the corresponding plots in the left column. (A–L) Assuming the network is infinitely stiff, deformable filaments push against either a flat surface (A–F) or a spherical surface (G–L). (A–C, G–I) All filaments are pushing ($k_a = 0$). (D–F, J–L) Filament attachments are included. (M–R) With a deformable network and attachments, filaments push against either a flat surface (M–O) or a spherical surface (P–R).

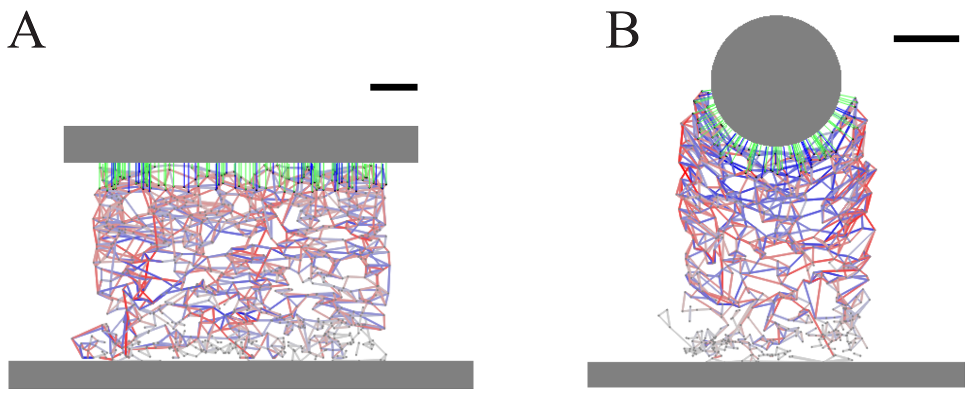

Figure S16. Simulation snapshots corresponding to Figure 4 A and B in the main text. Network springs are shown as colored lines with red representing compressed links and blue representing stretched links. Active filaments are lines perpendicular to the obstacle surface with green being free filaments and blue being attached filaments. Plates and bead are shown in gray. Bars: 1 μm.

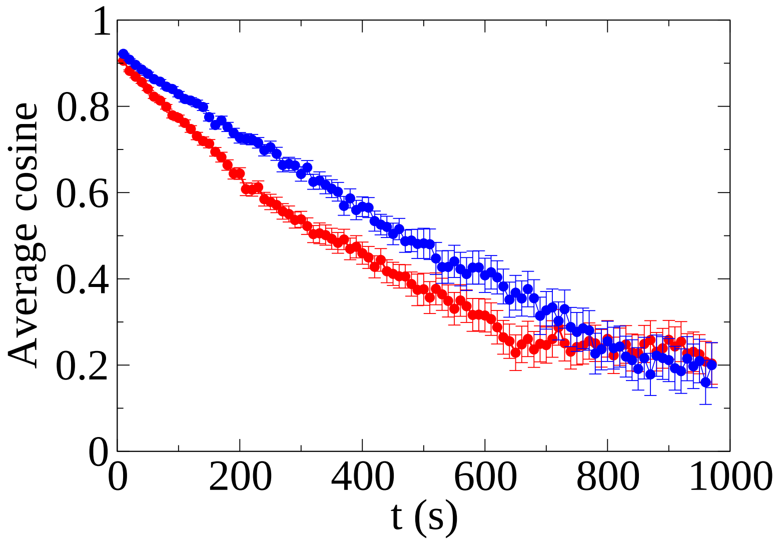

Figure S17. Observed average cosine value of the angle at which ellipsoidal beads move at given time. The angle is measured from beads' initial directions as a function of time. Red: beads moving along their long-axes. Blue: beads moving along their short-axes. The average cosine is close to zero when the beads make roughly a quarter of a circle. Knowing respective time and average linear speed, we estimate the average trajectory curvature to be about 0.1 μm. Note, that beads moving along their long axes curve faster; these beads also move with a slower linear speed. Thus, their trajectory curvature is higher.
